# FTY720 in resistant human epidermal growth factor receptor 2-positive breast cancer

**DOI:** 10.1038/s41598-021-04328-y

**Published:** 2022-01-07

**Authors:** Wei-Pang Chung, Wei-Lun Huang, Wei-An Liao, Chun-Hua Hung, Chi-Wu Chiang, Chun Hei Antonio Cheung, Wu-Chou Su

**Affiliations:** 1 Institute of Clinical Medicine, College of Medicine, National Cheng Kung University, Tainan, Taiwan; 2 Department of Oncology, National Cheng Kung University Hospital, College of Medicine, National Cheng Kung University, Tainan, Taiwan; 3 Center of Applied Nanomedicine, National Cheng Kung University, Tainan, Taiwan; 4 Department of Medical Laboratory Science and Biotechnology, College of Medicine, National Cheng Kung University, Tainan, Taiwan; 5 Department of Pathology, National Cheng Kung University Hospital, College of Medicine, National Cheng Kung University, Tainan, Taiwan; 6 Institute of Molecular Medicine, College of Medicine and Center for Infectious Disease and Signaling Research, National Cheng Kung University, Tainan, Taiwan; 7 Department of Pharmacology, College of Medicine, National Cheng Kung University, Tainan, Taiwan; 8 Institute of Basic Medical Sciences, College of Medicine, National Cheng Kung University, Tainan, Taiwan

**Keywords:** Cancer, Diseases, Medical research, Oncology

## Abstract

The prognosis of patients with human epidermal growth factor receptor 2 (HER2)-positive breast cancer has considerably improved. However, no reliable treatment besides anti-HER2 strategies has been available. FTY720, a small-molecule compound used for treating refractory multiple sclerosis, has been reported to have beneficial effects against cancers. We therefore evaluated the efficacy of FTY720 in trastuzumab-resistant breast cancer cells and investigated the possible mechanism involved. This study evaluated morphological changes after FTY720 treatment. Antiproliferative WST-1 assays and LDH Cytotoxicity Assay Kits were used to determine the treatment effects of drugs, whereas Western blot analysis was used to evaluate protein expression. Apoptotic events were investigated through annexin V staining and TUNEL assays using flow cytometry. FTY720 was effective in trastuzumab-resistant breast cancer cell lines despite the presence of *PIK3CA* mutation. Studied on a xenograft mouse model, FTY720-treated groups had statistically significantly poorer HCC1954 xenograft growth in vivo compared with the control group. Our findings suggest that FTY720 can overcome resistance to trastuzumab therapy in patients with HER2-positive breast cancer, with FTY720 plus trastuzumab might offer even better efficacy in vitro and in vivo.

## Introduction

Breast cancer is currently the leading type of malignancy among the female population worldwide. Approximately 20% of patients with breast cancer exhibit gene amplification and/or overexpression of human epidermal growth factor receptor 2 (HER2)^1^, which carries poorer prognosis in terms of risk for relapse and metastasis. Nonetheless, the development of trastuzumab for HER2-positive breast cancer has improved disease-free, progression-free, and overall survival^2–4^. Other targeted drugs, like lapatinib, trastuzumab emtansine, and pertuzumab, have also contributed to the success of HER2-directed treatments^5–8^. However, over 25% of patients with early stage breast cancer still suffer from recurrence after adjuvant trastuzumab treatments^9,10^. Moreover, patients with advanced-stage breast cancer will eventually be unable to control their disease despite receiving continuous HER2-directed therapies^2,5,7,8^. Several hypotheses have been proposed to address the mechanism of resistance, including HER2 downregulation, downstream gene mutation, or activation of other signaling pathways^11^. Indeed, drugs targeting phosphoinositide 3-kinase (PI3K) and/or mammalian target of rapamycin (mTOR) have shown promising results against resistance to HER2-targeted therapies in breast cancer cell lines^12^. Although one phase 3 study demonstrated that adding everolimus to second-line treatment significantly prolonged progression-free survival of patients with HER2-positive breast cancer^13^, the adverse effects of this combination therapy limited its adoption into general practice. Currently, no treatment guideline or proper strategy has been designed to overcome resistance to anti-HER2 therapies based on these proposed theories.

FTY720 (fingolimod) is a small-molecule compound that acts as a functional antagonist to the sphingosine-1-phosphate receptor. The drug has been approved for treating relapsing–remitting multiple sclerosis through its ability to prevent T-cell migration from lymph nodes^14,15^. Nowadays, FTY720 has been proven to have the ability to inhibit multiple intracellular targets, suggesting its potential role in cancer treatment^16^. FTY720 has also been shown to induce prominent apoptosis in cancer cell lines^17^ and has been reported to be an effective drug in several subtypes of breast cancer. Moreover, FTY720 in the combination with tamoxifen may overcome resistance in hormonal therapy-resistant breast cancer by inhibiting sphingosine-1-phosphate receptors or mimicking histone deacetylase inhibitors^18,19^. Together with epidermal growth factor receptor kinase inhibitors, FTY720 has shown its potential effects in inhibiting the growth of basal-like breast cancer cells^20^. FTY720 plus doxorubicin also shows its efficacy in sensitive HER2-positive breast cancer and resistant triple-negative breast cancer^21^. Despite showing promising effects in the treatment of breast cancer, the role of FTY720 in resistance to HER2-targeted therapies has yet to be investigated.

This study hypothesized that FTY720 can inhibit the proliferation of trastuzumab-resistant breast cancer cells through its multipotent anti-cancer effects. The effects of FTY720 were examined with or without trastuzumab across three trastuzumab-resistant breast cancer cell lines. This study opted to evaluate the effects of FTY720 in combination with trastuzumab in order to prevent compensatory mechanisms^22^. The possible mechanisms of cell death were also investigated. We then confirmed the efficacy of FTY720 in vivo using a heterotopic xenograft mouse model.

## Results

### Characterization of HER2-positive breast cancer cells

**Figure 1:**
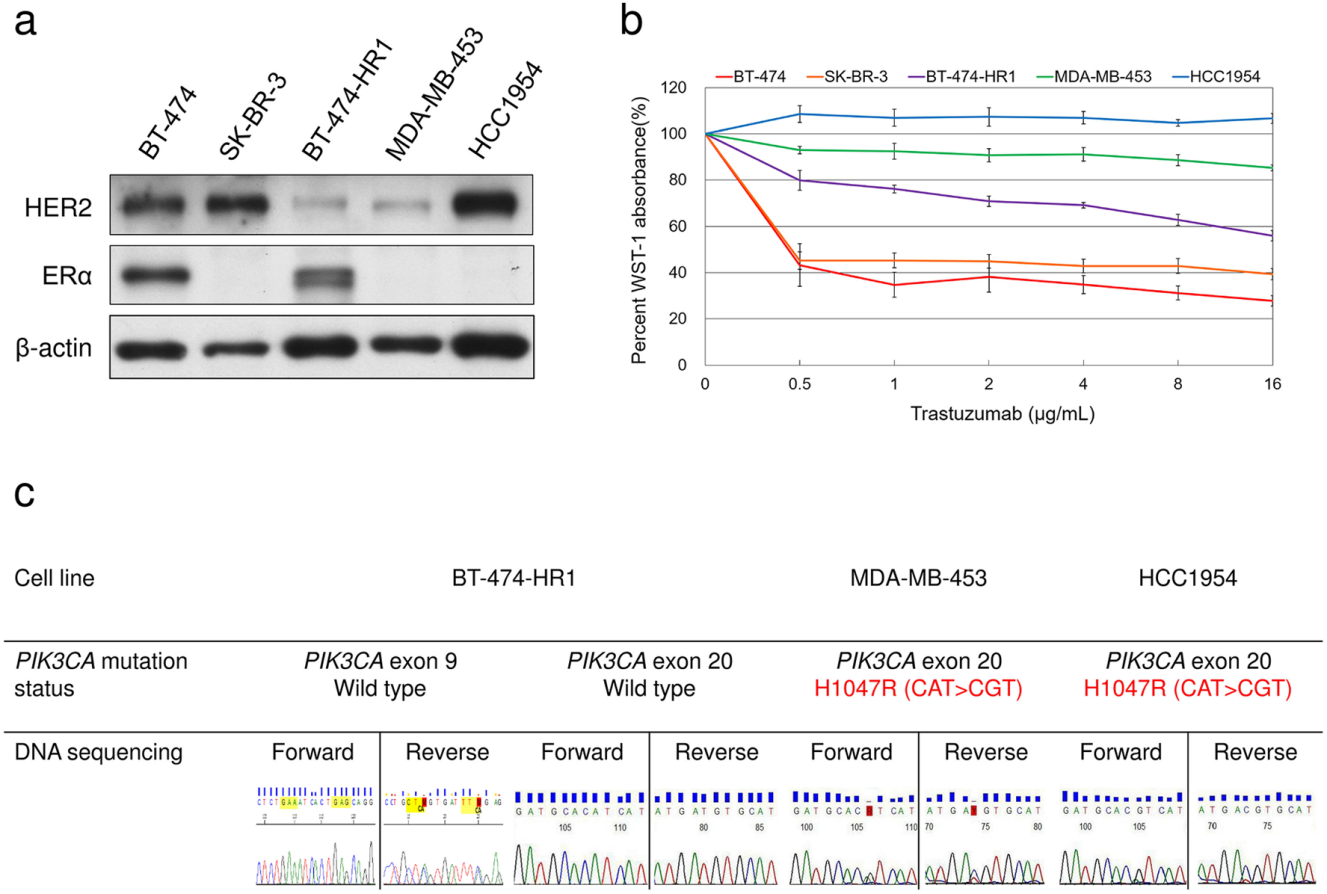
Characteristics of the five HER2-positive cell lines. (**a**) HER2 expression was confirmed through Western blot analysis in the five cell lines. Estrogen receptor expression was revealed in the BT-474 cell line compatible with its known feature. BT-474-HR1 cells derived from BT-474 cells retained estrogen receptor expression after selection from trastuzumab-resistant clones. (**b**) The growth inhibition status in the five cell lines was shown after treatment with different concentrations of trastuzumab. The percentage of WST-1 absorbance in the cells was determined 72 h after the incubation with trastuzumab (0.5–16 μg/mL) and then normalized to non-treated cells. Each plot indicates the mean value of at least three experiments, whereas error bars indicate the standard error of the mean. (**c**) The presence of *PIK3CA* gene mutation was evaluated in the three trastuzumab-resistant cell lines using direct exon 9 and 20 sequencing.

Five HER2-positive breast cancer cell lines were used herein. Apart from HER2 protein overexpression, BT-474 and BT-474-HR1 cells were also characterized by estrogen receptor expression (Fig. 1a) unlike the other three cell lines. BT-474-HR1 and MDA-MB-453 cells showed relatively low HER2 protein expression. We confirmed their HER2 gene amplification by conducting HER2 FISH in BT-474-HR1 and MDA-MB-453 cells. The former cell line showed a HER2 copy number of 25.50 and a HER2/CEP17 ratio of 6.89. The later cell line showed a HER2 copy number of 7.05 and a HER2/CEP17 ratio of 2.04 (Figure S1). BT-474 and SK-BR-3 cells were considered trastuzumab sensitive^23,24^, whereas MDA-MB-453 and HCC1954 cells were reported to be trastuzumab-resistant^12,22^. These cells were treated with trastuzumab at concentrations ranging from 0.5 to 16 μg/mL, showing different responses (Fig. 1b). PI3K mutations were the most frequent alterations detected among trastuzumab-resistant populations. Therefore, the three trastuzumab-resistant cell lines, BT-474-HR1, MDA-MB-453, and HCC1954, were sequenced for *PIK3CA* gene mutations (Fig. 1c). *PIK3CA* mutation in exon 20 (H1047R) was detected in MDA-MB-453 and HCC1954 cells, whereas no such mutation was detected in exons 9 or 20 in BT-474-HR1 cells. These results implicate a possibly different resistance mechanism in BT-474-HR1 cells compared with MDA-MB-453 and HCC1954 cells, which contained mutations in the catalytic domain of *PIK3CA*. Although BT-474 cells were reported to contain *PIK3CA* mutations in exon 2 (K111N), we still considered BT-474 as a *PIK3CA* wild-type cell line based on a previous review^25^. Table 1 summarizes the characteristics of all cells utilized with items including estrogen receptor, HER2, *PIK3CA* gene, and sensitivity to trastuzumab.

**Table 1:** Characteristics of the five breast cancer cell lines.

Cell lines	ER protein	HER2 protein	*PIK3CA* gene	IC_50_ level of trastuzumab
BT-474	Expression	Overexpression	Wild type	< 0.5 μg/mL
SK-BR-3	Non-expression	Overexpression	Wild type	< 0.5 μg/mL
BT-474-HR1	Expression	Overexpression	Wild type	> 16 μg/mL
MDA-MB-453	Non-expression	Overexpression	Exon 20 H1047R mutation	> 16 μg/mL
HCC1954	Non-expression	Overexpression	Exon 20 H1047R mutation	> 16 μg/mL

ER, estrogen receptor; HER2, human epidermal growth factor receptor 2.

### FTY720 is effective against trastuzumab-resistant breast cancer cells and triggers programmed cell death

**Figure 2:**
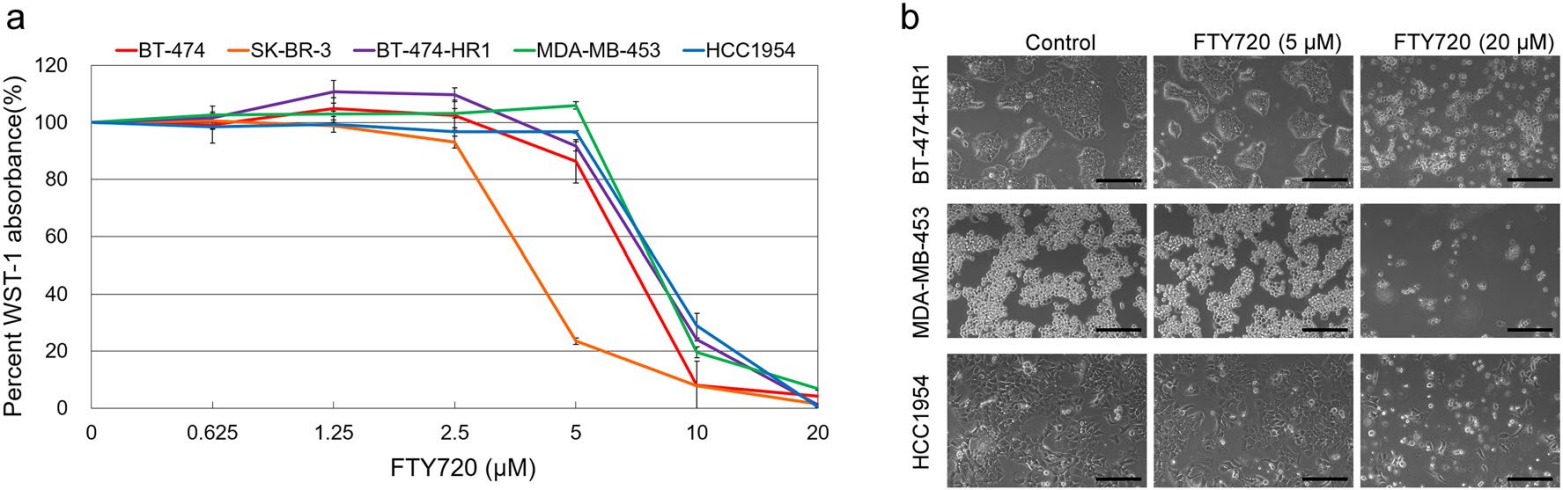
FTY720 hinders the growth of trastuzumab-resistant cells. (**a**) FTY720 inhibited cell growth in the five HER2-overexpressing cell lines. The percentage of WST-1 absorbance in cells was measured 72 h after the incubation with FTY720 (0.625–20 μM) with results being normalized to cells without treatment. Each plot indicates the mean value of at least three experiments, whereas error bars indicate the standard error of the mean. (**b**) The morphology of the three trastuzumab-resistant cell lines was analyzed using a light microscope after FTY720 treatment at 0, 5, and 20 μM. All experiments were repeated at least three times. Bar, 200 μm.

Two trastuzumab sensitive and three trastuzumab-resistant cell lines were treated with FTY720 at concentrations ranging from 0.625 to 20 μM (Fig. 2a). Possible IC_50_ values were between 5 and 10 μM for BT-474 cells and 2.5 and 5 μM for SK-BR-3 cells. These results were similar to previously published data^21^. For the three trastuzumab-resistant lines, IC_50_ values were all between 5 and 10 μM. The morphology of BT-474-HR1, MDA-MB-453, and HCC1954 cells was determined after FTY720 treatment at concentrations of 0, 5, and 20 μM (Fig. 2b). FTY720 inhibited cell growth at 20 μM, though not much effect was observed at 5 μM. These findings correlated with the results of the antiproliferative assay. Moreover, cytoplasmic vacuolization and loss of integrity were observed in the cells after FTY720 treatment at the effective concentration.

**Figure 3:**
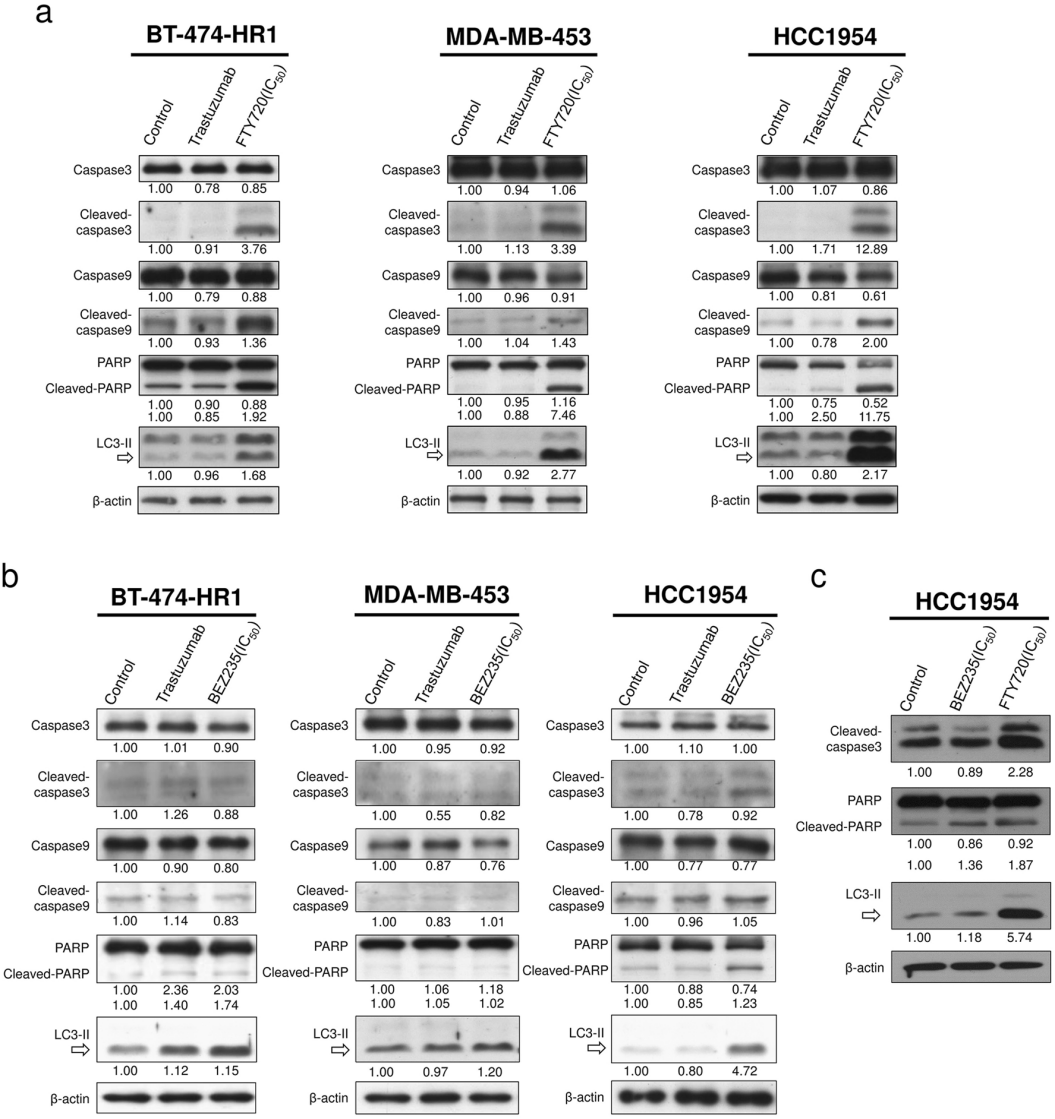
FTY720 induces apoptotic and autophagic signaling that is more potent than that of BEZ235. (**a**) Lysates were collected 24 h after trastuzumab or FTY720 treatment and then subjected to immunoblotting with the indicated antibodies. (**b**) Lysates were collected and evaluated 24 h after trastuzumab or BEZ235 treatment. (**c**) Protein expressions with BEZ235 and FTY720 were compared at IC_50_ 24 h after treatment in HCC1954 cells. FTY720 concentrations were 7.5, 7.5, and 10 μM for BT-474-HR1, MDA-MB-453, and HCC1954, respectively. BEZ235 concentrations were 16, 16, and 64 nM for BT-474-HR1, MDA-MB-453, and HCC1954, respectively. Equal loading of lysates was verified using the amount of beta-actin. Numbers under each Western blot represent the intensity of the protein relative to that of the control. All experiments were repeated at least three times.

Considering that the presence of cytoplasmic vacuolization has been discussed in certain types of cell death^26^ and FTY720 did induce apoptosis of mouse breast cancer cells^17^, we examined proteins involved in apoptosis and autophagy. Lysates of three trastuzumab-resistant cell lines were collected 24 h after mock, trastuzumab, or FTY720 treatment. Our results showed prominent overexpression of cleaved caspase-3, cleaved caspase-9, cleaved PARP, and LC3-II after FTY720 treatment (Fig. 3a, Figure S5a). Adding FTY720 has been suggested to turn on programmed cell death in these cells. We substituted BEZ235, a dual PI3K and mTOR inhibitor bearing the ability to induce apoptosis in breast cancer cells^27^, for FTY720 in the aforementioned protocols to determine whether the same trend for protein expression existed (Fig. 3b, Figure S5b). BEZ235 treatment upregulated the expression of cleaved caspase-3, cleaved caspase-9, cleaved PARP, and LC3-II. However, protein expression did not seem as prominent as that induced by FTY720. Both drugs were tested in HCC1954 cells at IC_50_ on the same panel. Accordingly, FTY720 induced greater apoptotic and autophagic signaling compared with BEZ235 in terms of increased expression of cleaved caspase-3, cleaved PARP, and LC3-II (Fig. 3c).

**Figure 4:**
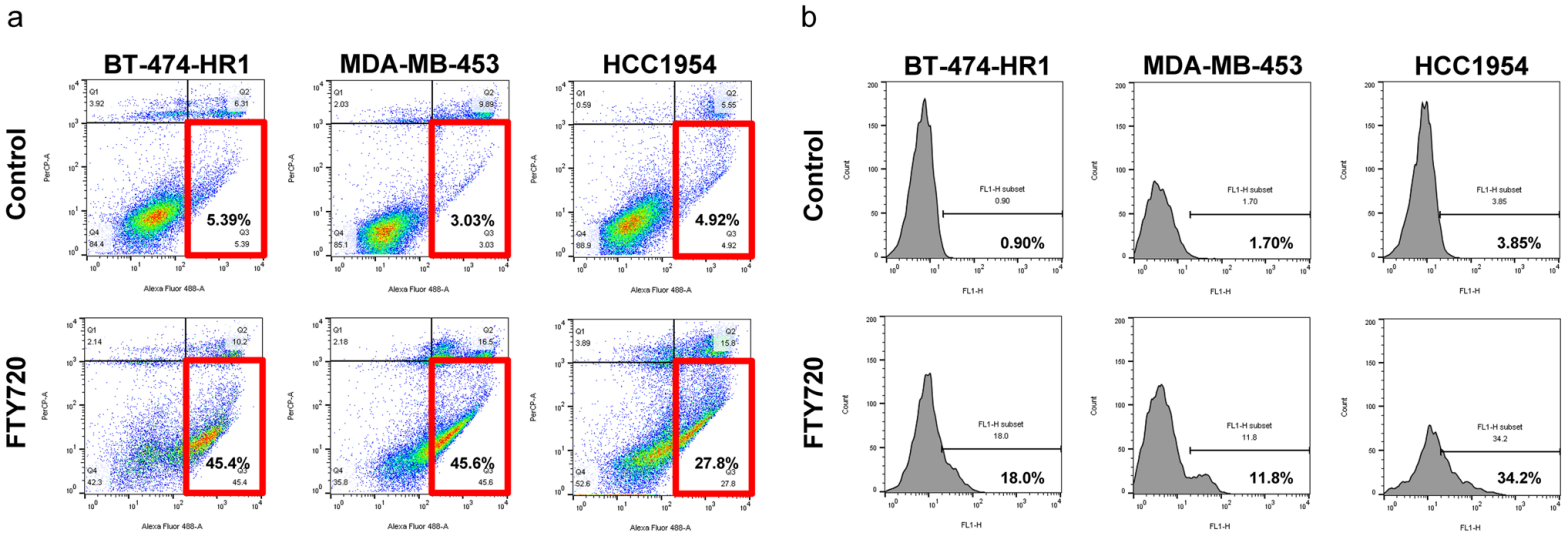
FTY720 triggers apoptotic events in trastuzumab-resistant cell lines. (**a**) The three trastuzumab-resistant cell lines were treated with DMSO only as control or FTY720 at IC_50_ for 24 h. Cells were then stained with annexin V and analyzed using flow cytometry to determine the proportion of apoptotic cells. The x-axis indicates Alexa Fluor 488-A, whereas the y-axis indicates PerCP-A. (**b**) TUNEL assay kits were utilized to compare the percentage of apoptotic DNA fragmentation after DMSO and FTY720 treatment at IC_50_. The incubation time was 32 h for BT-474-HR1 and 40 h for MDA-MB-453 and HCC1954. The x-axis indicates FL1-H, whereas the y-axis indicates cell count.

We further validated FTY720-induced apoptotic events using two methods. Cells were stained with annexin V and analyzed using flow cytometry (Fig. 4a, Figure S5c). The percentage of apoptotic cells increased substantially in the three trastuzumab-resistant cell lines after incubation with FTY720 for 24 h. Accordingly, the percentage of apoptotic cells after mock treatment was 5.39%, 3.03%, and 4.92% in BT-474-HR1, MDA-MB-453, and HCC1954 cells, which increased to 45.4%, 45.6%, and 27.8% after FTY720 treatment, respectively. These results were confirmed by incubating HCC1954 cells with BEZ235. Accordingly, our results showed apoptotic trends similar to those with FTY720 (Figure S2). However, the percentage of apoptotic cells was lower in HCC1954 cells compared with that in the other two cell lines. We hypothesized that FTY720 might not have reached its maximum effect in HCC1954 cells. Therefore, we attempted exposing HCC1954 cells to a FTY720 concentration higher than IC_50_ or prolonging the incubation time with FTY720 from 24 to 48 h. Both of adjustments contributed to a higher percentage of apoptotic cells (Figure S3), suggesting differences in the peak reaction timing among cells. Furthermore, TUNEL assays were used to detect apoptotic DNA fragmentation. All three trastuzumab-resistant cell lines demonstrated increased DNA fragmentation after exposure to FTY720 (Fig. 4b, Figure S5c). The percentage of DNA fragmentation increased from 0.90 to 18.0%, 1.70 to 11.8%, and 3.85 to 34.2% in BT-474-HR1, MDA-MB-453, and HCC1954 cells, respectively. Prominent apoptotic events were also be demonstrated by increased sub-G0 cells after adding FTY720 (Figure S4).

### FTY720 potentiates death of resistant cells through concurrent apoptotic pathway activation and autophagic pathway inhibition

**Figure 5:**
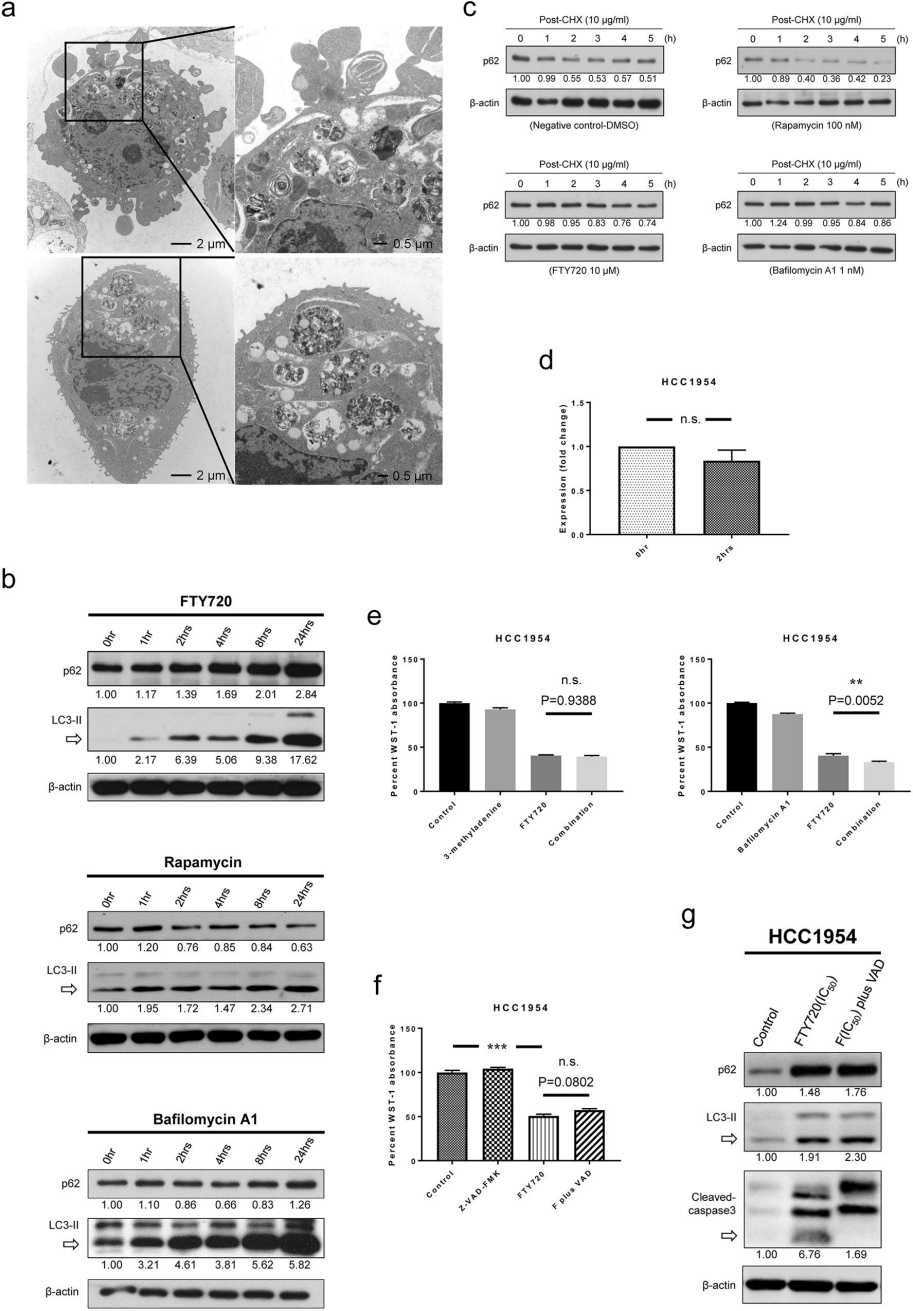
FTY720 overcomes resistance to trastuzumab by influencing the regulation of apoptosis and autophagy. (**a**) HCC1954 cells were collected and prepared 24 h after FTY720 treatment. Cell morphology was assessed using an electron microscope. (**b**) Autophagy-related proteins, p62 and LC3-II, were analyzed after HCC1954 cells were treated with FTY720, rapamycin, and bafilomycin A1. (**c**) The protein stability test was performed using cycloheximide chase assays after treatment with the indicated drugs in HCC1954 cells. Lysates were collected every hour up to 5 h after cycloheximide treatment. (**d**) mRNA levels of *p62* in HCC1954 cells were evaluated using reverse transcription quantitative real-time PCR 0 and 2 h after FTY720 incubation. (**e**) Cells were treated with 3-methyladenine or bafilomycin A1 with or without FTY720. (**f**) Cells were treated with the pan caspase inhibitor Z-VAD-FMK, FTY720, or a combination of both drugs. In the combination group, cells were pretreated with Z-VAD-FMK for 1 h followed by FTY720 treatment. (**g**) Lysates were collected and analyzed 24 h after FTY720 treatment. In the combination group, cells were treated with Z-VAD-FMK 1 h before FTY720. Numbers under cleaved caspase-3 section indicate the intensity of the blot pointed by the arrow. All experiments were repeated at least three times except for images captured using the electron microscope. During Western blot analysis, equal loading of proteins was verified using beta-actin, and numbers under each Western blot indicate the intensity of the protein relative to that at 0 h or of the control. During evaluation of antiproliferative effects, the percentage of WST-1 absorbance in cells was measured 72 h after drug incubation. Each plot indicates the mean value, whereas error bars indicate the standard error of the mean. The concentrations of FTY720, rapamycin, bafilomycin A1, 3-methyladenine, and Z-VAD-FMK were 10 μM, 100 nM, 1 nM, 20 μM, and 20 μM, respectively. *P *value: **P < 0.01 and ***P < 0.001. CHX: cycloheximide, F plus VAD: FTY720 plus Z-VAD-FMK, n.s.: not significant.

An electron microscope was utilized to determine intracellular morphological changes in affected cells. HCC1954 cells treated with 20 µM FTY720 were collected and prepared 24 h later. These cells contained multiple folded layers of membranes and devoured organelles (Fig. 5a). Such structures were similar to autophagosomes surrounding a portion of the cytoplasm as described in the literature^28^ and were implicated in the autophagic process. Lysates of FTY720-treated HCC1954 cells were also collected. P62 and LC3-II expressions increased with exposure time, a finding similar to that for bafilomycin A1-treated cells but not rapamycin-treated cells (Fig. 5b, Figure S6a). This is because autophagy inhibitors, such as bafilomycin A1, promote p62 accumulation, whereas autophagy inducers, such as rapamycin, gradually decrease p62 expression. Protein stability tests for p62 were then performed after FTY720, bafilomycin A1, or rapamycin treatment in HCC1954 cells. Following cycloheximide addition 3 h after exposure to the target drugs, protein translation was halted. Protein analysis was performed based on the scheduled timing. Trends in p62 stability were similar between FTY720- and bafilomycin A1-treated cells but again different from rapamycin-treated cells (Fig. 5c, Figure S6b). The expression of *p62* messenger RNA in FTY720-treated HCC1954 cells was then examined using PCR. Fold changes in messenger RNA between 0 and 2 h did not significantly differ (Fig. 5d). The aforementioned findings suggested that FTY720-mediated changes in p62 protein expression were independent of protein translation. To confirm the role of FTY720 as an autophagy inhibitor, HCC1954 cells were co-treated with FTY720 and a known autophagy inhibitor to determine whether the antiproliferative effects of FTY720 could be restored. After co-treatment with 3-methyladenine and bafilomycin A1, our results showed that the FTY720-mediated antiproliferative effects were not restored by other autophagy inhibitors (Fig. 5e). We also elucidated the effects of apoptosis inhibition on FTY720-mediated antiproliferation. After HCC1954 cells were co-treated with FTY720 and one pan caspase inhibitor, Z-VAD-FMK, the FTY720-mediated antiproliferative effects were not restored (Fig. 5f). Through Western blot analysis, the cleavage of caspase-3 was halted by adding Z-VAD-FMK 1 h before FTY720 treatment. Accumulation of caspase-3 fragments with high molecular weight was observed, which could indicate blockage of the caspase-dependent pathway^29–31^. Within the same events, increased expression of p62 and LC3-II was noted after apoptotic pathway blockage (Fig. 5g). This suggested that autophagic rescue could not restore cell death following the effects of FTY720 treatment, which not only triggers apoptosis but also concurrently inhibits the autophagic pathway.

### FTY720 in combination with trastuzumab provides better outcomes against trastuzumab-resistant HER2-positive breast cancer cells in vitro and in vivo

**Figure 6:**
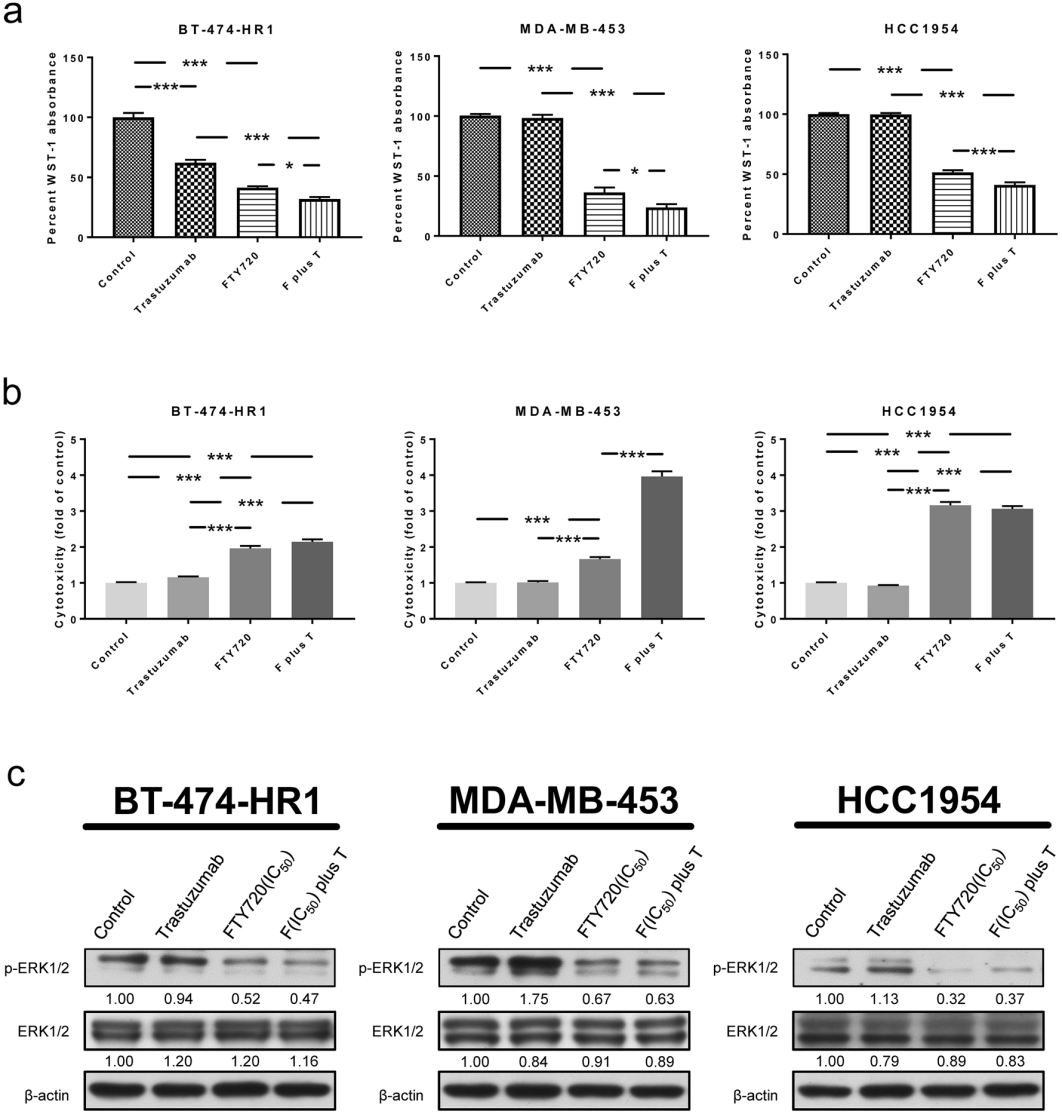
Effects of FTY720 in breast cancer cell lines, alone or in combination with trastuzumab. (**a**) The three cell lines were treated with DMSO, trastuzumab, FTY720, or FTY720 plus trastuzumab. The percentage of WST-1 absorbance was measured 72 h after treatment. (**b**) Cytotoxicity was determined using LDH‐cytotoxicity assay kits 72 h after treatment. The cytotoxicity index was calculated, whereas the value of the control group treated with DMSO was adjusted to 1. (**c**) Lysates were collected and analyzed for phospho-ERK1/2, ERK1/2, and beta-actin 24 h after treatment. Equal loading of proteins was verified using beta-actin. Numbers under each blot indicate the intensity of the blot relative to the control. The concentration of trastuzumab used in all cell lines was 2 μg/mL. The concentrations of FTY720 were 7.5, 7.5, and 10 μM for BT-474-HR1, MDA-MB-453, and HCC1954, respectively. Regarding antiproliferative and cytotoxic effects, results were presented as mean ± SEM, and expresses were repeated at least three times. *P *value: *P < 0.05, **P < 0.01, and ***P < 0.001. F plus T: FTY720 plus trastuzumab.

Given the importance of the HER2 signaling pathway in HER2-positive breast cancer, HER2-directed therapies have still been the primary strategy employed to deal with progressive disease after trastuzumab treatment^5,7,13^. We examined whether adding trastuzumab could potentiate the effects of FTY720. Trastuzumab was dosed consistently at 2 μg/mL in each group and cell line, whereas the dosage of FTY720 was adjusted according to the IC_50_ in each cell line. The results showed that co-treatment with FTY720 and trastuzumab significantly increased the antiproliferative effects compared with monotherapy (Fig. 6a). We used similar designs and utilized LDH‐Cytotoxicity Assay Kits to measure cytotoxic effects. Accordingly, adding trastuzumab significantly potentiated the cytotoxic effects of FTY720 in MDA-MB-453 cells. Although adding trastuzumab did not significantly potentiate the effects of FTY720 in BT-474-HR1 and HCC1954 cells, FTY720 by itself was still able to provide considerable cytotoxic effects (Fig. 6b). The aforementioned results suggested that continuous blockage in HER2-dependent signaling pathway was still beneficial despite failure of HER2-directed therapies. We examined protein expression after FTY720 and trastuzumab treatment. Notable downregulation of phospho-ERK was observed after FTY720 treatment in the three resistant cell lines (Fig. 6c, Figure S6c). There was no effect of trastuzumab on the expression of phopho-ERK but the presence of FTY720 caused statistically significant down-regulation of phopho-ERK in certain cell lines. The negative regulation of phospho-ERK might have been attributed to the opposing effects of protein phosphatase 2A (PP2A) activation^32,33^, with studies recognizing FTY720 as a PP2A activator^21,34^.

**Figure 7:**
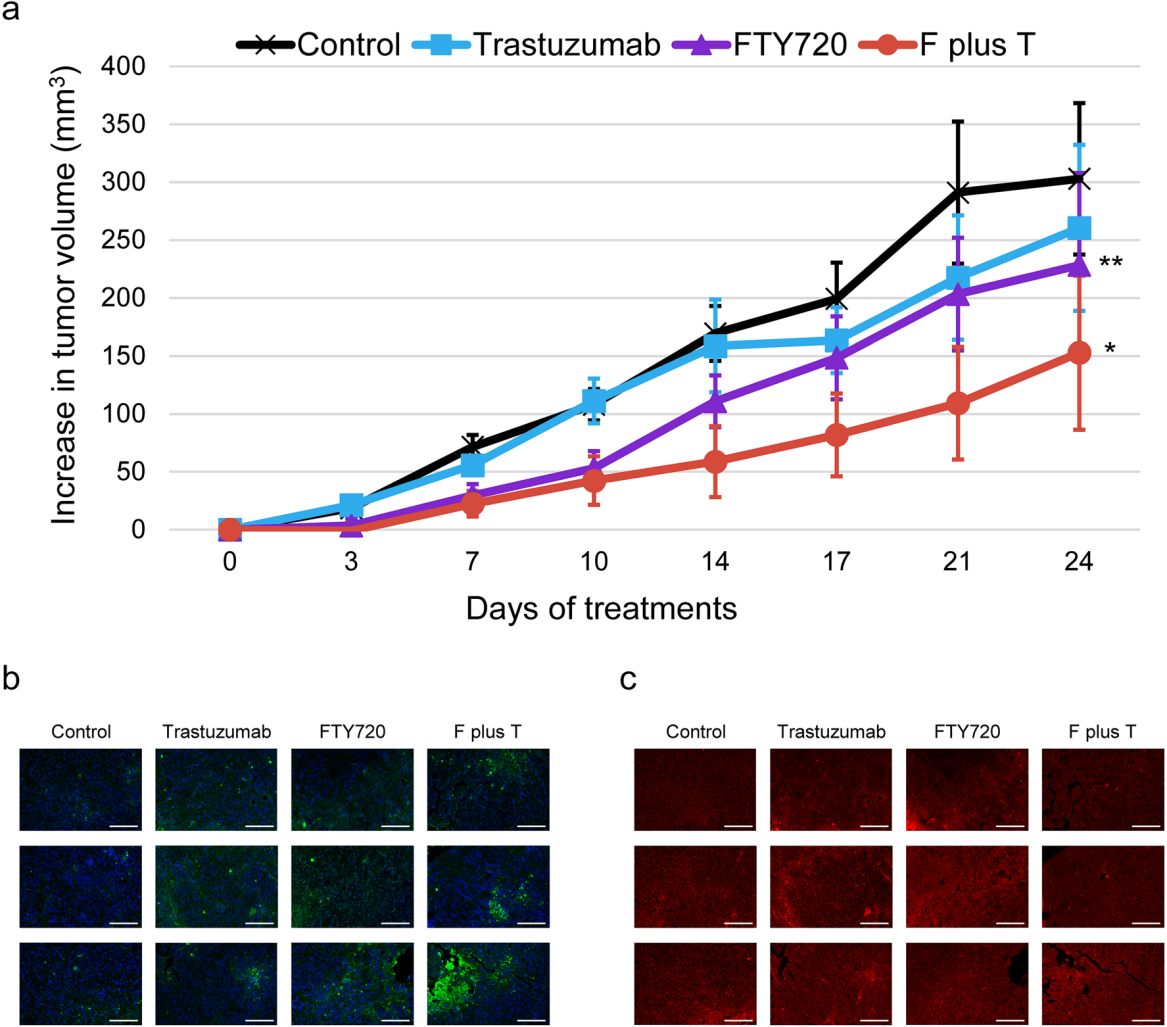
Xenograft mouse model of HCC1954 cells showing the efficacy of FTY720 with or without trastuzumab. (**a**) Mice bearing HCC1954 xenografts were randomized into four treatment groups (n = 9 for each group) when tumor size reached 150–200 mm^3^. Mice were treated with PBS, trastuzumab, FTY720, or FTY720 plus trastuzumab. Xenografts were harvested 24 h after the last treatment. Each plot indicates the mean increase in tumor volume from the first day of treatment, whereas error bars indicate the standard error of the mean. Differences between the four groups were analyzed using ANOVA with mixed-effect model. Differences in control versus FTY720 and control versus FTY720 plus trastuzumab were statistically significant. *P *value: *P < 0.05 and **P < 0.01. (**b**) Tumor tissue sections were evaluated using TUNEL assays (green) and also counterstained with Hoechst 33,342 dye (blue, indicating nuclei). They were then examined using fluorescence microscopy. The top three tissue sections with the most abundant green signals in each group were presented. (**c**) Using the same tissue sections, cell membranes were outlined using HER2-antibody conjugating secondary antibody (red) and then examined using fluorescence microscopy. Bar, 100 μm. F plus T: FTY720 plus trastuzumab.

We then investigated the in vivo effects of FTY720 by establishing a xenograft mouse model. Accordingly, HCC1954 cells were inoculated into BALB/c nude mice, which were then randomly assigned to four treatment groups: control, trastuzumab, FTY720, and FTY720 plus trastuzumab. Treatments were administered via intraperitoneal injection for 23 days, whereas the concentrations of each drug used are detailed in the “Heterotopic xenograft mouse model and in situ TUNEL assays” section. The trastuzumab group had modestly poorer tumor growth compared with the control group (Fig. 7a). In contrast, the FTY720 group had significantly poorer xenograft growth compared with the control group (*P* = 0.0093) and so did the FTY720 plus trastuzumab group compare with the control group (*P* = 0.0253). Two mice died during the treatment period, one in the control group and the other in the trastuzumab group.

Xenografts were then harvested and prepared as tissue sections. To determine drug-induced apoptotic events in vivo, tumor slides were stained with Hoechst 33,342 dye followed by TUNEL assays and observed using fluorescence microscopy. Results showed prominent green signaling indicating apoptotic events in FTY720-treated groups (Fig. 7b). In the FTY720 plus trastuzumab group, obvious apoptotic events were still observed even though these xenografts were much smaller than those in other three groups. Some green signal was not accompanied by blue stains indicating the presence of a nucleus. To determine whether false positive stains existed, the same tissue sections were stained with HER2 antibody outlining the structure of the membranes and then examined using fluorescence microscopy (Fig. 7c). Within areas without DAPI stains, we confirmed the co-existence between green and red signals, proving that these were not false positive apoptotic events.

## Discussion

FTY720, which serves as an immunosuppressive agent, has been approved for the treatment of relapsing–remitting multiple sclerosis. Nonetheless, its ability to confer antineoplastic effects has been gradually discovered. From hematologic malignancies to several types of solid tumors, FTY720 has shown its potential role in anti-cancer treatments in vitro and in vivo through several postulated mechanisms^34–38^. The present study demonstrated that FTY720 could overcome resistance to trastuzumab in HER2-positive breast cancer cells. FTY720 induced prominent apoptotic events in trastuzumab-resistant breast cancer cells. Most importantly, FTY720 acted as an autophagy inhibitor in resistant cells and further potentiated its cytotoxic effects. Moreover, the combination of FTY720 and trastuzumab offered more potent effects than FTY720 alone based on our analysis of antiproliferative activity and cytotoxicity. The treatment effects of FTY720 plus trastuzumab had also been validated in a xenograft mouse model.

Patients with HER2-positive breast cancer carrying PI3K mutations have been shown to have poorer prognosis^39^. Although PI3K/AKT/mTOR pathway activation contributes toward resistance to anti-HER2 treatment, drugs that offer significant clinical benefits have still been lacking. BEZ235, a potent dual PI3K and mTOR inhibitor, has been proven effective in trastuzumab-resistant cells^40^. Unfortunately, this drug has been abandoned because of its toxicity. The present study validated the efficacy of BEZ235 in three trastuzumab-resistant cell lines. Accordingly, protein analysis showed that BEZ235 induced not only apoptosis but also overexpression of autophagy-related proteins. However, after exposure to BEZ235 and FTY720 at IC_50_, more prominent overexpression of cleaved caspase-3, cleaved PARP, and LC3-II was observed with FTY720. This suggests that FTY720 better promotes apoptosis- or autophagy-related cell death. Unlike BEZ235, FTY720 has had acceptable toxicity profiles among humans. Moreover, the FTY720 dose selected in our mouse model (25 mg/kg/week) did not exceed those reported in multiple sclerosis or solid tumor animal models (ranged from 7 to 70 mg/kg/week)^21,35,41,42^. These properties make FTY720 a potential candidate for HER2-positive human breast cancer trials.

FTY720 induced prominent apoptotic signaling in trastuzumab-resistant cells, which was confirmed through Western blot analysis, annexin V staining followed by flow cytometry, and TUNEL assays. Previous studies had also shown that FTY720 contributed to the apoptosis of mouse breast cancer cells identified through electron microscopy^17^. Here, we utilized electron microscopy to determine apoptotic features in HCC1954 cells 24 h after FTY720 treatment. However, instead of typical features indicating apoptosis, our results revealed autophagy-associated structures, such as multiple folded layers of membranes and devoured organelles. Though such a result does not conclude the absence of apoptotic events, it may imply the various timings required to observe different kinds of programmed cell death. In fact, the findings captured by the electron microscopy did suggest that FTY720 was an autophagy modulator in trastuzumab-resistant breast cancer cells. Studies have shown that FTY720 acts mostly as an autophagy inducer in other types of cancer cells^38,43^. Therefore, we thoroughly examined the role of FTY720 in autophagy among HCC1954 cells. Similar to the other autophagy inhibitor, bafilomycin A1, exposure to FTY720 increased the expression of p62 and LC3-II with exposure time. This result definitely differed from that in HCC1954 cells treated with rapamycin, a well-known autophagy inducer. Besides, p62 protein stability analysis showed that changes in p62 expression after FTY720 treatment were similar to those with bafilomycin A1 but not rapamycin. Moreover, after co-treating HCC1954 cells with FTY720 and one autophagy inhibitor, our results showed that the antiproliferative effects of FTY720 in such cells could not be restored by adding the autophagy inhibitor. Notably, cell growth still could not be restored by adding a pan caspase inhibitor that works against FTY720-induced apoptosis in trastuzumab-resistant breast cancer cells. In some conditions, apoptotic events were not reversed by blockage of the caspase-dependent pathway given that the cell survival mechanism was shunted toward the autophagy-dependent pathway^44^. This phenomenon was confirmed by the even greater overexpression of p62 and LC3-II after adding the pan caspase inhibitor to HCC1954 cells treated with FTY720. Given the ability to trigger apoptosis and inhibit autophagic pathways, FTY720 could block the escape mechanism of cells, which depends on autophagy during the activation of apoptosis. This led to prominent cell death even in trastuzumab-resistant cells carrying the notorious *PIK3CA* mutation. The crosstalk between apoptosis and autophagy may contribute to the resistance of anti-HER2 therapies in HER2-positive breast cancer^45^. For a drug inducing apoptosis and inhibiting autophagy, this is a rationale that we should elucidate the efficacy of FTY720 further in humans.

Continuous blockage of HER2 protein with anti-HER2 monoclonal antibodies still plays an important role in patients whose diseases continue to progress after anti-HER2 therapies^46^. Again, this was confirmed in vitro and in vivo by adding trastuzumab to FTY720. Accordingly, our results showed that FTY720 plus trastuzumab was more efficient than FTY720 alone in most settings in terms of antiproliferative effects, cytotoxic effects, and the ability to control tumor growth. These effects could have been attributed to trastuzumab-related HER2 blockage and FTY720-mediated blockage of intracellular signaling activated by other tyrosine kinase receptors. The dual blockade more thoroughly inhibited the growth of tumor cells. In the xenograft mouse model, FTY720 alone provided significantly greater inhibition of HCC1954 xenograft growth compared with the control (PBS). FTY720 plus trastuzumab might even provide better effects than FTY720 alone in terms of mean tumor size reduction. However, this difference did not reach statistically significant (*P* = 0.0824), and it may probably be affected by the limited numbers of mice. Limited effects were observed in the trastuzumab-alone group, which might have been due to the antibody-dependent cell-mediated cytotoxicity (ADCC) of trastuzumab^47^. All these results highlight the benefit of combining FTY720 and trastuzumab for trastuzumab-resistant breast cancer. This combination regimen was also well tolerated in the mouse model considering that no death event was recorded in the combination group during the 4 week treatment period.

FTY720 has been reported to mediate immunosuppression via inhibiting lymphocyte emigration from lymphoid organs^15^. This may raise the concern about the combination strategy with FTY720 and trastuzumab. Since ADCC is mainly dependent on innate immune cells such as NK cells, neutrophils, and macrophages^48,49^ but not T lymphocytes, the FTY720-mediated immunosuppression will have little impact on ADCC. In addition, in our xenograft animal study using BALB/cAnN.Cg-*Foxn1*^*nu*^/CrlNarl mice with the lack of T cells, we could still observe ADCC effects that are similar to the previous publication^47^. It is believed that the ADCC induced by anti-HER2 monoclonal antibodies in humans should be more prominent. Therefore, we believe FTY720 together with anti-HER2 monoclonal antibodies may provide the best chance for this drug to be developed in human studies.

## Conclusion

The present study showed that FTY720 possesses the ability to overcome resistance to trastuzumab therapy in HER2-positive breast cancer with or without *PIK3CA* mutation. Through effects involving apoptosis and autophagy, FTY720 alone or in combination with trastuzumab caused death in different trastuzumab-resistant breast cancer cells with FTY720 plus trastuzumab offering the best efficacy. Considering that FTY720 has been used for treating refractory multiple sclerosis in humans, its safety profiles have been well established. Thus, our results suggest that FTY720 can be considered as a potential drug to be developed in early phase clinical trials for patients with HER2-positive breast cancer whose diseases are resistant to trastuzumab.

## Materials and methods

### Cell lines, cell culture, and reagents

BT-474 (RRID:CVCL_0179) cells were obtained from Bioresource Collection and Research Center (BCRC, Taiwan) and maintained in Hybri-Care medium [American Type Culture Collection (ATCC), Manassas, VA, USA] with 10% fetal bovine serum (FBS) (Gibco by Life Technologies, Waltham, MA, USA). SK-BR-3 (RRID:CVCL_0033) cells were obtained from ATCC and maintained in McCoy's 5A Medium (Merck KGaA, Darmstadt, Germany) with 10% FBS. HCC1954 (RRID:CVCL_1259) cells were obtained from ATCC and maintained in RPMI-1640 medium (Gibco by Life Technologies) with 10% FBS. MDA-MB-453 (RRID:CVCL_0418) cells were obtained from BCRC and maintained in Leibovitz's L-15 Medium (Merck KGaA) with 10% FBS. The trastuzumab-resistant cell line, BT-474-HR1, was established by continuous selection from parental BT-474 cells after exposure to gradually increasing doses of trastuzumab with concentrations higher than the IC_50_ of the parental BT-474 cells. Thereafter, BT-474-HR1 cells were routinely maintained with trastuzumab at a concentration of 64 μg/mL. All cell lines were maintained at 37 °C in 5% carbon dioxide, except for MDA-MB-453 cells that were maintained at 37 °C without carbon dioxide supplementation. All experiments were performed with mycoplasma-free cells. All cell lines have been authenticated using short tandem repeat profiling within the last three years. Trastuzumab, which was purchased from the pharmacy at National Cheng Kung University Hospital, was manufactured by Genentech (San Francisco, CA, USA) and diluted with phosphate-buffered saline (PBS). FTY720 was purchased from Luminescence Technology Corporation (Taiwan) and prepared with dimethyl sulfoxide (DMSO). BEZ235 and Z-VAD-FMK were purchased from Selleck Chemicals (Houston, TX, USA) and were both prepared with DMSO. Cycloheximide, bafilomycin A1, rapamycin, and 3-methyladenine were purchased from Merck KGaA and prepared with DMSO.

### HER2 fluorescence in situ hybridization (FISH)

Cells were harvested by trypsinization, fixed with formalin, and embedded using paraffin for slide preparation. Formalin-fixed paraffin–embedded samples were then cut into 4 μm sections and placed on slides. These samples were further dehydrated by a xylene washing step followed by 100% ethanol. Slides were incubated with 0.2 N hydrochloric acid followed by distilled water wash then incubated 8–10 min with VP2000 protease solution (Abbott, Abbott Park, IL, USA) followed by 5 min with pretreatment wash buffer. Dehydration process was performed by increasing ethanol concentration (70%, 85% and 100%). HER2 and chromosome enumeration probe 17 (CEP17) probes (PathVysion HER2 DNA Probe Kit II, Abbott) were hybridized in a wet chamber overnight at the temperature of 37 °C. Slides were washed with 2 × saline-sodium citrate buffer. We took images using fluorescence microscope (Axioskop 2, Zeiss, Oberkochen, Germany) after 4',6-diamidino-2-phenylindole counterstaining was conducted. Forty cells were collected for the analysis of HER2 copy number and HER2/CEP17 ratio. The HER2 amplification was interpreted based on the American Society of Clinical Oncology (ASCO)/College of American Pathologists (CAP) HER2 testing guideline in breast cancer^50^.

### *PIK3CA* gene sequencing

Exons 9 and 20 of the *PIK3CA* gene were analyzed through polymerase chain reaction (PCR) amplification of genomic DNA and direct sequencing of PCR products. Primers for *PIK3CA* exons 9 and 20 were as follows: exon 9: TTG CTT TTT CTG TAA ATC ATC T (forward) and CTG CTT TAT TTA TTC CAA TAG G (reverse); exon 20: CTC AAT GAT GCT TGG CTC TG (forward) and TGG AAT CCA GCG TGA GCT TTC (reverse). Sequencing was performed using an ABI 3500 Dx Genetic Analyzer.

### In vitro antiproliferative activity analysis

Cells were seeded at concentrations of 1 × 10^4^–3 × 10^4^ cells/200 μL/well in 96-well plates for 24 h and treated with indicated agents for 72 h. After the treatments, the WST-1 proliferation assay was performed according to the manufacturer's instructions. Briefly, 10 µL of WST-1 reagent (Takara Bio Inc., Kusatsu, Shiga, Japan) was added into each well and incubated for 1 h. Results were determined by measuring the absorbance of the solution at a wavelength of 450 nm using a spectrophotometer.

### In vitro apoptosis analysis

To detect apoptosis, both annexin V cell staining and TUNEL assays were used. For annexin V staining, cells were collected and resuspended in Annexin V Binding Buffer and stained with Annexin V-FITC and propidium iodide (BD, Franklin Lakes, NJ, USA) for 15 min at room temperature according to the manufacturer's instructions. Cells were then analyzed using flow cytometry (FACSCalibur, BD). For TUNEL assays, cells were fixed with 1% paraformaldehyde in PBS (4 °C, 30 min), washed in PBS, permeabilized with ice-cold 70% ethanol, and stained using APO-Direct TUNEL kits (BD) according to the manufacturer's instructions. Briefly, cells were incubated with the DNA-labeling solution (containing TdT enzyme and FITC-dUTP) for 2 h at 37 °C with occasional shaking, washed, and treated with PI/RNase staining buffer for 30 min at room temperature. Samples were then immediately analyzed using flow cytometry (FACSCalibur).

### Reverse transcription quantitative real-time PCR

Total RNA was extracted using the single-step TRIzol method (Thermo Fisher, Waltham, MA, USA) according to the manufacturer's protocol. For reverse transcription PCR, the cDNA was synthesized from 0.05 μg of total RNA using the Reverse Transcription System (Promega, Fitchburg, WI, USA). *P62* mRNA expression was measured through quantitative real-time PCR with SYBR green reagents (Thermo Fisher) using the StepOne Real-Time PCR System (Thermo Fisher). GAPDH gene expression was used as an endogenous control. Primers for GAPDH were AGGTC ATCCC TGAGC TGAAC GG (forward) and CGCCT GCTTC ACCAC CTTCT TG (reverse). Expression levels were calculated using the 2^−∆∆Ct^ ratio^51^. The following *p62* PCR primers were used (Genomics, Taiwan): GCA CCC CAA TGT GAT CTG C (forward) and CGC TAC ACA AGT CGT AGT CTG G (reverse).

### Western blot analysis

For cell lysis, harvested samples were incubated on ice in whole-cell-extract lysis buffer for 30 min. Lysates were then centrifuged at 12,000 rpm, 4℃, for 10 min, after which protein concentration was measured using the Bradford assay (Bio-Rad, Hercules, CA, USA). For Western blot analysis, 15–100 μg of lysates (depending on the targeted proteins) were boiled for 5 min with sample buffer before being separated on sodium dodecyl sulfate–polyacrylamide gels. Proteins were transferred to polyvinylidene difluoride membranes (Millipore, Billerica, MA, USA) and blocked with 5% nonfat milk/TBST buffer. Primary antibodies used were as follows: HER2, beta-actin (Merck KGaA), ERα, ERK1/2 (Santa Cruz Biotechnology, Santa Cruz, CA, USA), caspase-3, cleaved caspase-3, caspase-9, cleaved caspase-9, poly (ADP-ribose) polymerase (PARP) (Cell Signaling Technology, Beverly, MA, USA), p62, phospho-ERK1/2 (GeneTex, Irvine, CA, USA), and LC3 (MBL international, Woburn, MA, USA). Anti-PARP antibody can also recognize cleaved PARP proteins. Anti-rabbit and anti-mouse secondary antibodies were purchased from Jackson ImmunoResearch (West Grove, PA, USA). ImageJ software (National Institutes of Health, USA) was used to determine the intensity of each blot relative to the control after adjustment based on the intensity of beta-actin.

### Protein stability test

Protein stability was evaluated using cycloheximide chase assays. Briefly, cells were seeded for 24 h and treated with or without the indicated drugs, including FTY720, rapamycin, and bafilomycin A1, for another 3 h. The cells were then treated with cycloheximide with cell lysates being collected at indicated time points. p62 and beta-actin expressions were analyzed using Western blot analysis^52^.

### In vitro cytotoxicity analysis

BT-474-HR1, MDA-MB-453, and HCC1954 cells were seeded into 96‐well plates and treated with DMSO (as control), trastuzumab, FTY720, or trastuzumab plus FTY720. Cytotoxicity was determined using LDH‐Cytotoxicity Assay Kit II (Abcam, Cambridge, MA, USA) according to the manufacturer's instructions and quantified by measuring the absorbance of the solution at a wavelength of 450 nm using a spectrophotometer. The cytotoxicity index of each treatment group was calculated using the equation (test sample − low control) / (high control − low control). “Test sample” indicates the level of different groups; “low control” indicates the level of the reagent (minimal LDH‐value); and “high control” indicates the level of the DMSO group (maximal LDH‐value).

### Heterotopic xenograft mouse model and in situ TUNEL assays

Five- to six-week-old BALB/cAnN.Cg-*Foxn1*^*nu*^/CrlNarl mice were used and cared following the institutional guideline. To establish a xenograft mouse model, HCC1954 cells (3 × 10^6^) in 100 μL PBS were inoculated subcutaneously into the flank of each mouse. Tumor size was measured in two dimensions: length and width. Tumor volume was estimated using the equation (length × width^2^) / 2. When tumor sizes reached 150 to 200 mm^3^, mice were randomly allocated into four groups with each treatment group containing nine mice. All drugs were administered via intraperitoneal injection. The control group received PBS 5 days per week, whereas the other three groups received trastuzumab at 30 mg/kg/day twice per week, FTY720 at 5 mg/kg/day five times per week, and a combination of trastuzumab and FTY720, respectively. Tumor xenografts were measured for size (with calipers) and volume twice weekly. Following treatment for 23 days, mice were sacrificed by inhalation of carbon dioxide 24 h after the final administration. Tumors were harvested, and then further prepared as formalin-fixed/paraffin-embedded tissues. This animal study is reported in accordance with ARRIVE guidelines (https://arriveguidelines.org). The animal use protocol discussed previously was reviewed and approved by the Institutional Animal Care and Use Committee, National Cheng Kung University (106,108). Tumor tissue sections were stained with the TUNEL reagent using the Fluorescein (green) In Situ Apoptosis Detection Kit (Roche Diagnostics, Rotkreuz, Switzerland) according to the manufacturer's protocol. After counterstaining with Hoechst 33,342 dye (blue, staining nuclei, Merck KGaA) and HER2 antibody/PE-Texas Red-conjugated secondary antibody (red, plasma membrane, Merck KGaA), the cells were examined using fluorescence microscopy^53^.

### Data analysis and statistics

Each in vitro experimental design was repeated in triplicate at minimum to confirm replicability of results. Experimental results assessing the five cell lines under various treatment conditions were analyzed using unpaired t tests, and ANOVA when multiple comparisons were involved**.** These methods are loaded in GraphPad Prism version 9.2.0 for Windows (GraphPad Software, La Jolla CA, USA, www.graphpad.com). Each plot represents the mean value of the data, whereas the error bars on each plot represent the standard error of the mean (SEM). Growth curves of tumor xenografts showed the increase in tumor volume by days of treatment. Each plot represents the mean increase in tumor volume, whereas error bars represent the SEM. Differences between the four groups were analyzed using ANOVA with mixed-effect model embedded in GraphPad Prism version 9.2.0. *P* values less than 0.05 were regarded as statistically significant.

### Ethics approval

All animal handling was reviewed and approved by the Institutional Animal Care and Use Committee, National Cheng Kung University (106108).

## Supplementary Information

Supplementary Information 1.

Supplementary Information 2.
