# Supplementary material for: FTY720 in resistant human epidermal growth factor receptor 2-positive breast cancer
Source: Sci Rep. 2022 Jan 7;12:241. doi: 10.1038/s41598-021-04328-y (PMC8742024; doi:10.1038/s41598-021-04328-y)
Supplement: Supplementary file 1 — Supplementary Information 1. [file 41598_2021_4328_MOESM1_ESM.docx]

Figure S1. **HER2 gene amplification identified by FISH.** (a) BT-474-HR1 and (b) MDA-MB-453 cells both showed amplification of HER2 gene. Orange signals were HER2 genes and green signals were chromosome 17 centromeres.

Figure S2. **Both FTY720 and BEZ235 can induce apoptotic events in HCC1954 cells.** HCC1954 cells were treated with FTY720 or BEZ235 at IC_50_. After 24 h, the cells were stained with annexin V and analyzed using flow cytometry to evaluate the proportion of apoptotic cells. The x-axis indicates Alexa Fluor 488-A, whereas the y-axis indicates PerCP-A.

Figure S3. **FTY720-induced apoptotic events vary with higher dosages and longer incubation times.** FTY720-induced apoptotic events in HCC1954 cells were examined 24 h after incubation at 12.5 μM or 48 h after incubation at 10 μM. Cells were stained with annexin V and analyzed using the flow cytometry to determine the percentage of apoptotic cells. The x-axis indicates Alexa Fluor 488-A, whereas the y-axis indicates PerCP-A.

Figure S4. **FTY720 increases DNA fragmentation.** DNA fragmentation was determined in the three trastuzumab-resistant cell lines with or without FTY720 treatment. Cells were tested using the TUNEL assay kits and further analyzed using flow cytometry. Red circles contained defined DNA fragments. The x-axis indicates FL2-A, whereas the y-axis indicates FL1-H.

Figure S5. **Quantification and statistical analysis of Western blot analysis and apoptotic events.** (a, b) The intensity of cleaved-caspase3, cleaved-caspase9, cleaved-PARP, and LC3-II was quantitated relative to that of the control. (c) The percentage of apoptotic cells and the percentage of DNA fragmentation in FTY720-treated cells have been compared to the control ones. *P*-value: **P* < 0.05, ***P* < 0.01, and ****P* < 0.001.

Figure S6. **Quantification and statistical analysis of Western blot analysis.** (a) The expression of p62 and LC3-II after treatments with FTY720, rapamycin, and bafilomycin A1 was quantitated. (b) The expression of p62 after treatments with DMSO, FTY720, rapamycin, and bafilomycin A1 was quantitated. (c) The expression of phosphor-ERK1/2 was quantitated and statistically analyzed after the treatments with indicated drugs. *P*-value: **P* < 0.05, ***P* < 0.01.
