# Supplementary material for: FTY720 in resistant human epidermal growth factor receptor 2-positive breast cancer
Source: Sci Rep. 2022 Jan 7;12:241. doi: 10.1038/s41598-021-04328-y (PMC8742024; doi:10.1038/s41598-021-04328-y)
Supplement: Supplementary file 2 — Supplementary Information 2. [file 41598_2021_4328_MOESM2_ESM.pdf]

Figure S1

BT-474-HR1 MDA-MB-453

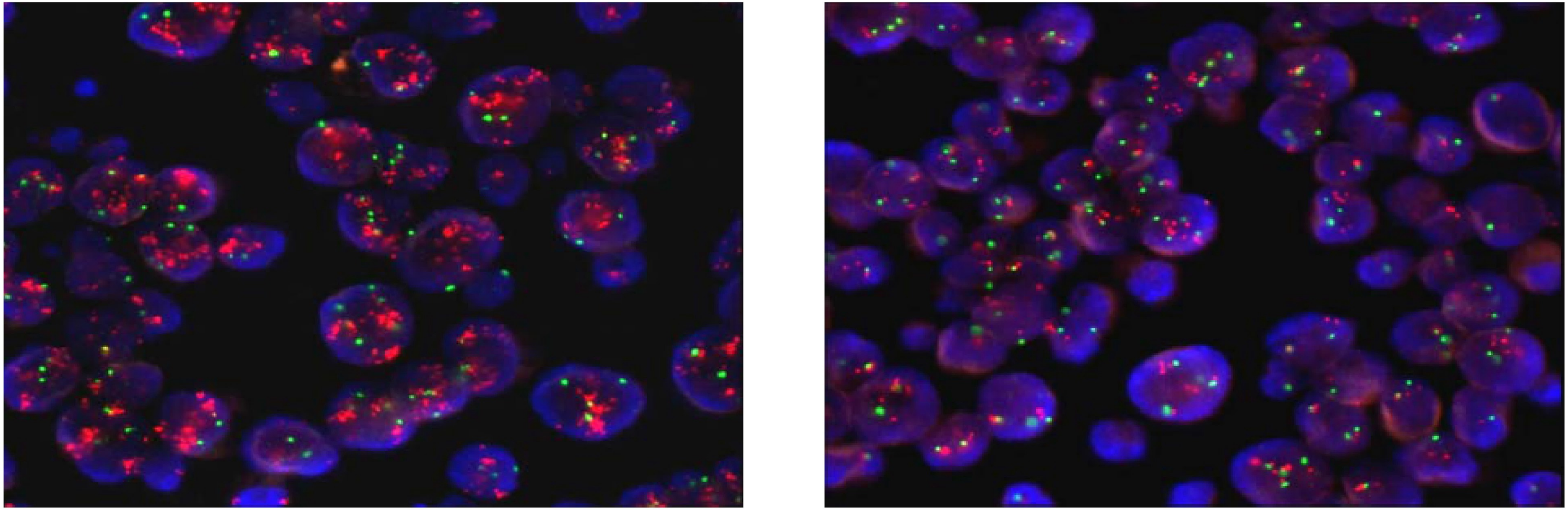

Figure S2

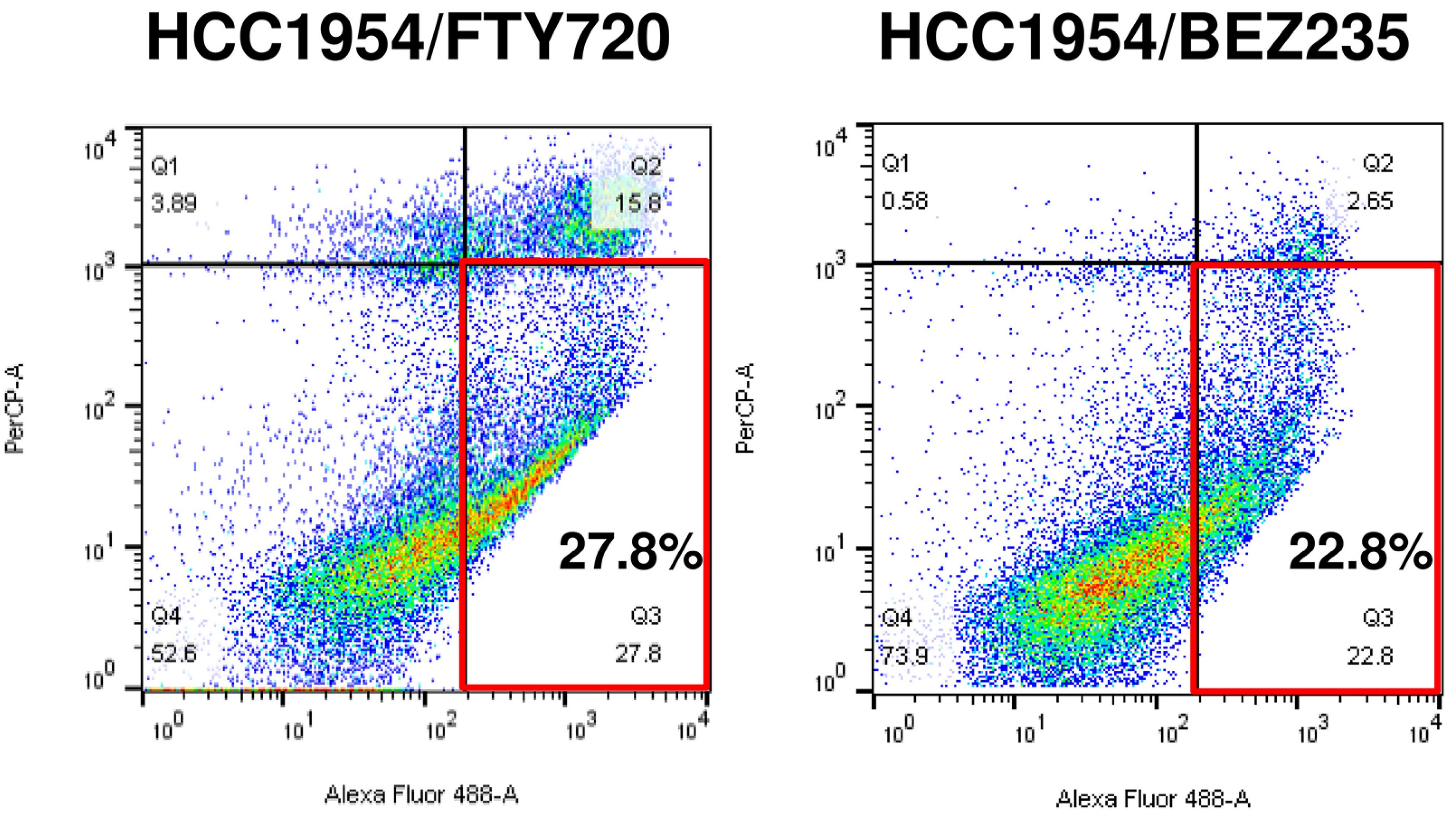
HCC1954/FTY720
HCC1954/BEZ235
Q1
3.89
Q2
15.8
Q4
52.6
Q3
27.8
27.8%
Q1
0.58
Q2
2.65
Q4
73.9
Q3
22.8
22.8%
PerCP-A
Alexa Fluor 488-A

Figure S3

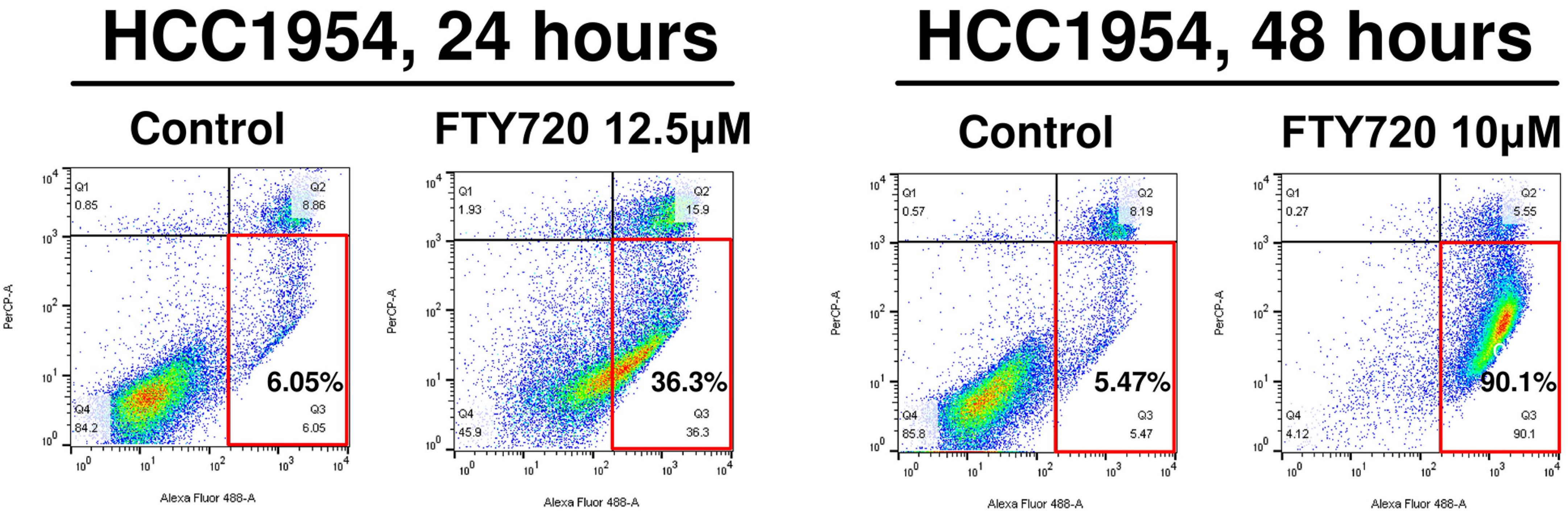
HCC1954, 24 hours
HCC1954, 48 hours
Control
FTY720 12.5μM
Control
FTY720 10μM
Q1 0.85
Q2 8.86
Q4 84.2
Q3 6.05
6.05%
Q1 1.93
Q2 15.9
Q4 45.9
Q3 36.3
36.3%
Q1 0.57
Q2 8.19
Q4 85.8
Q3 5.47
5.47%
Q1 0.27
Q2 5.55
Q4 4.12
Q3 90.1
90.1%
PerCP-A
Alexa Fluor 488-A

Figure S4

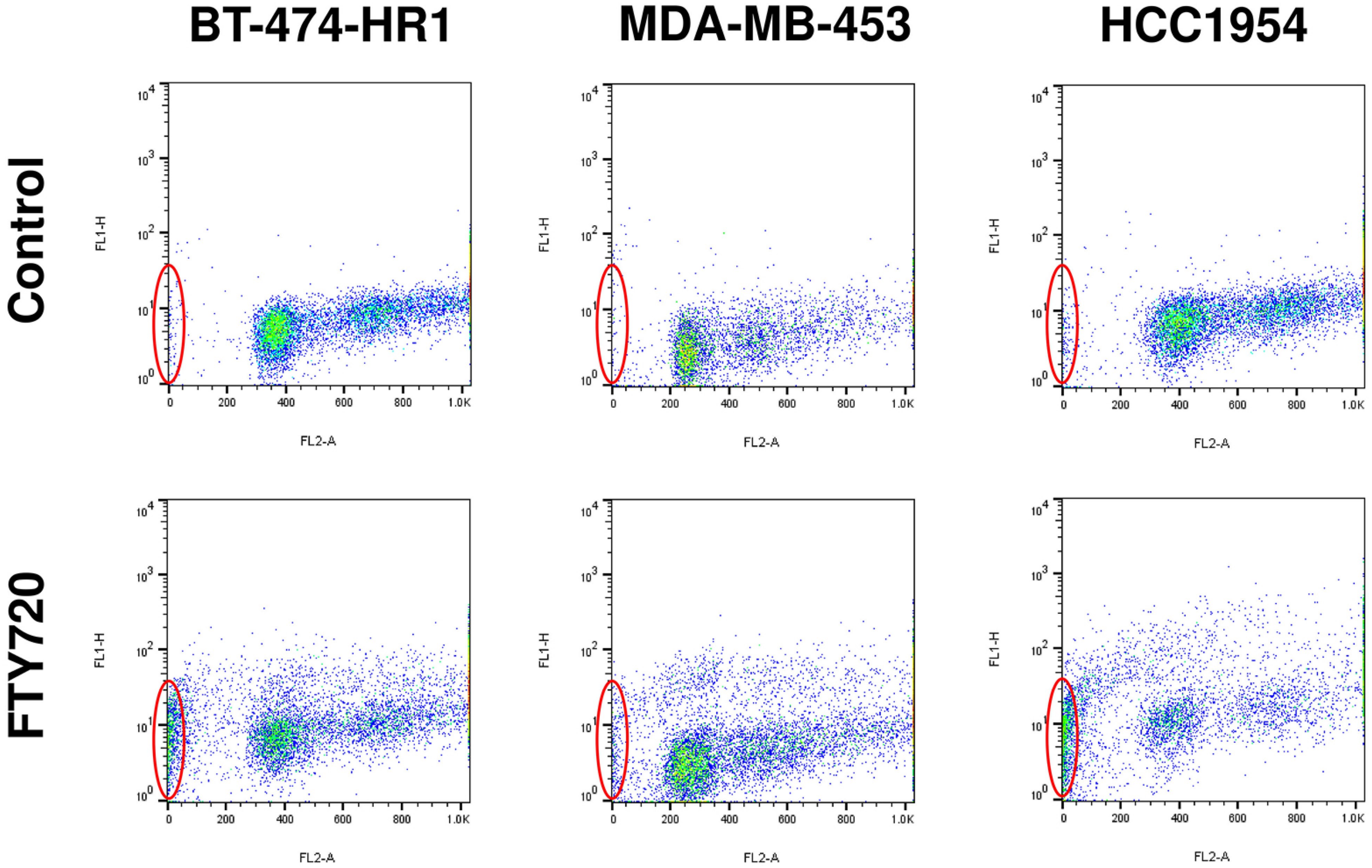

BT-474-HR1
MDA-MB-453
HCC1954
Control
FTY720
FL1-H
FL2-A
10^0
10^1
10^2
10^3
10^4
0
200
400
600
800
1.0K

Figure S5

a

Figure 3a

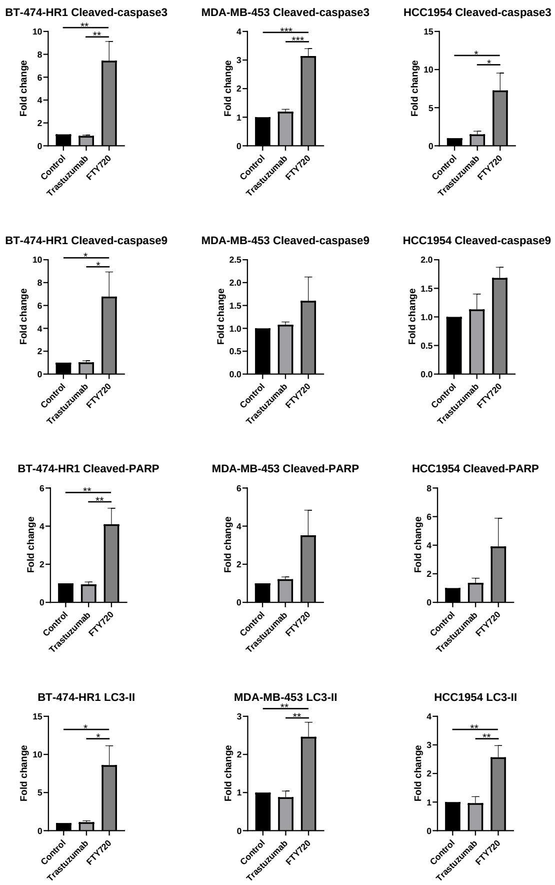

b

Figure 3b

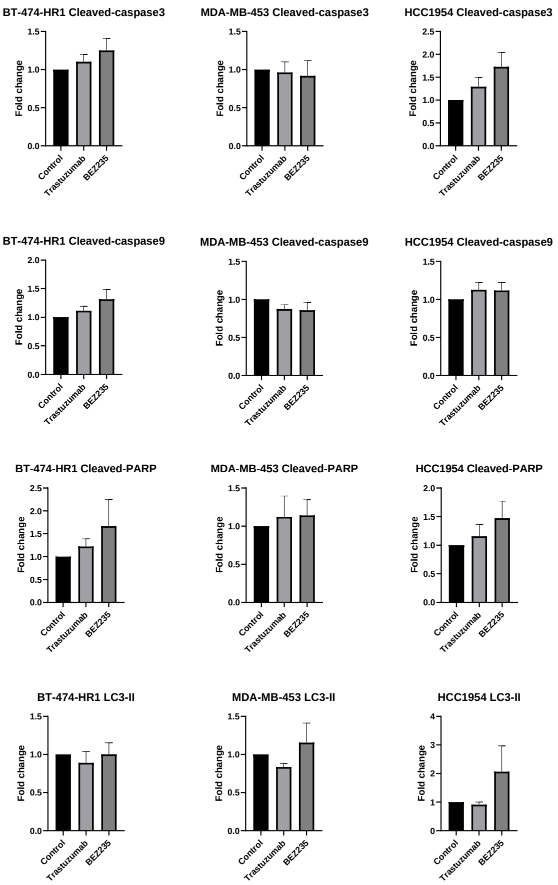

c

Figure 4a

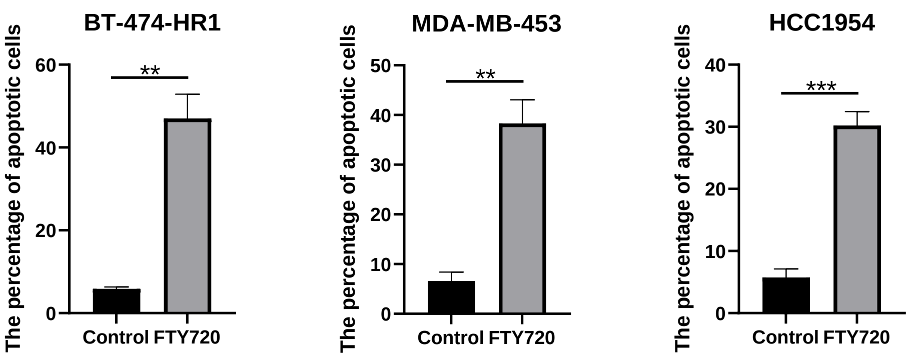

Figure 4b

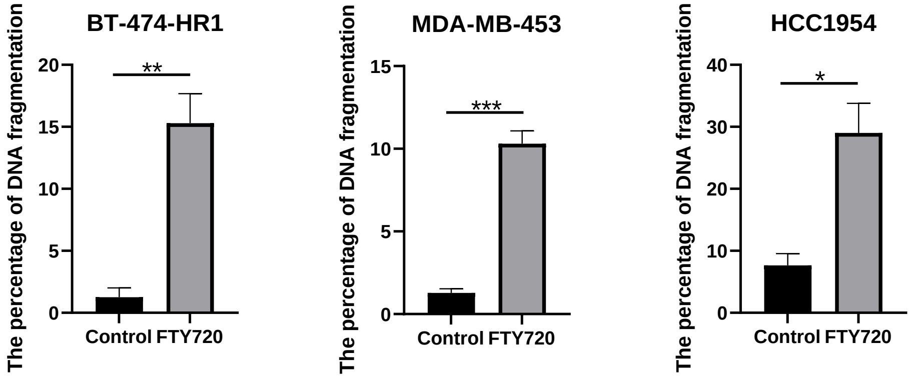

Figure S6

a Figure 5b

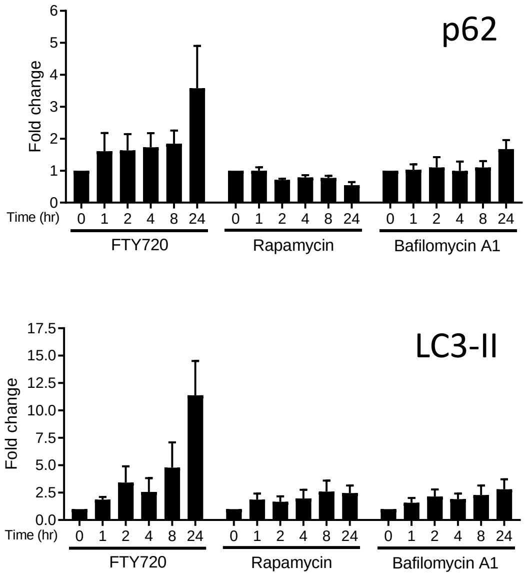

b Figure 5c

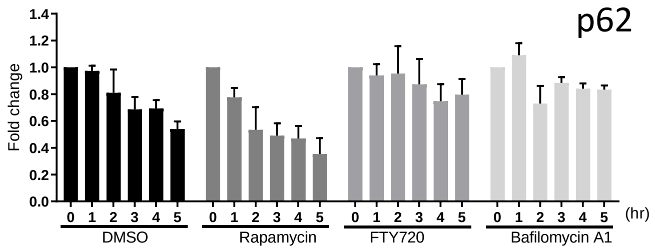

c Figure 6c

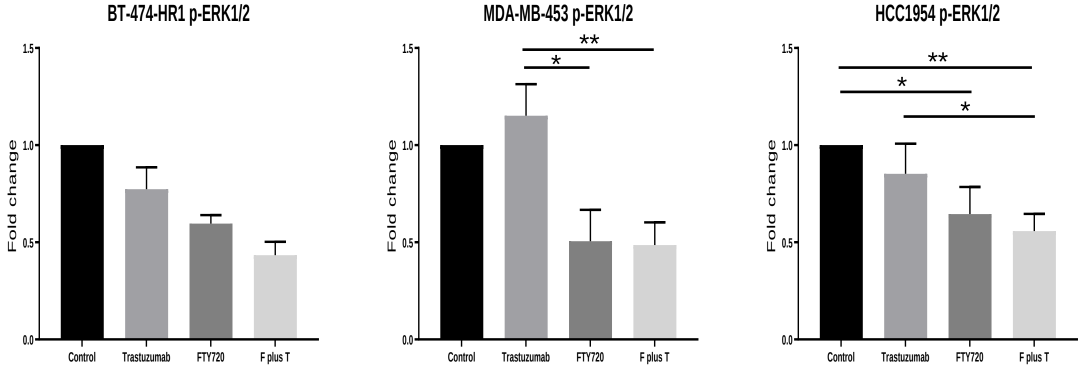

Figure 1a

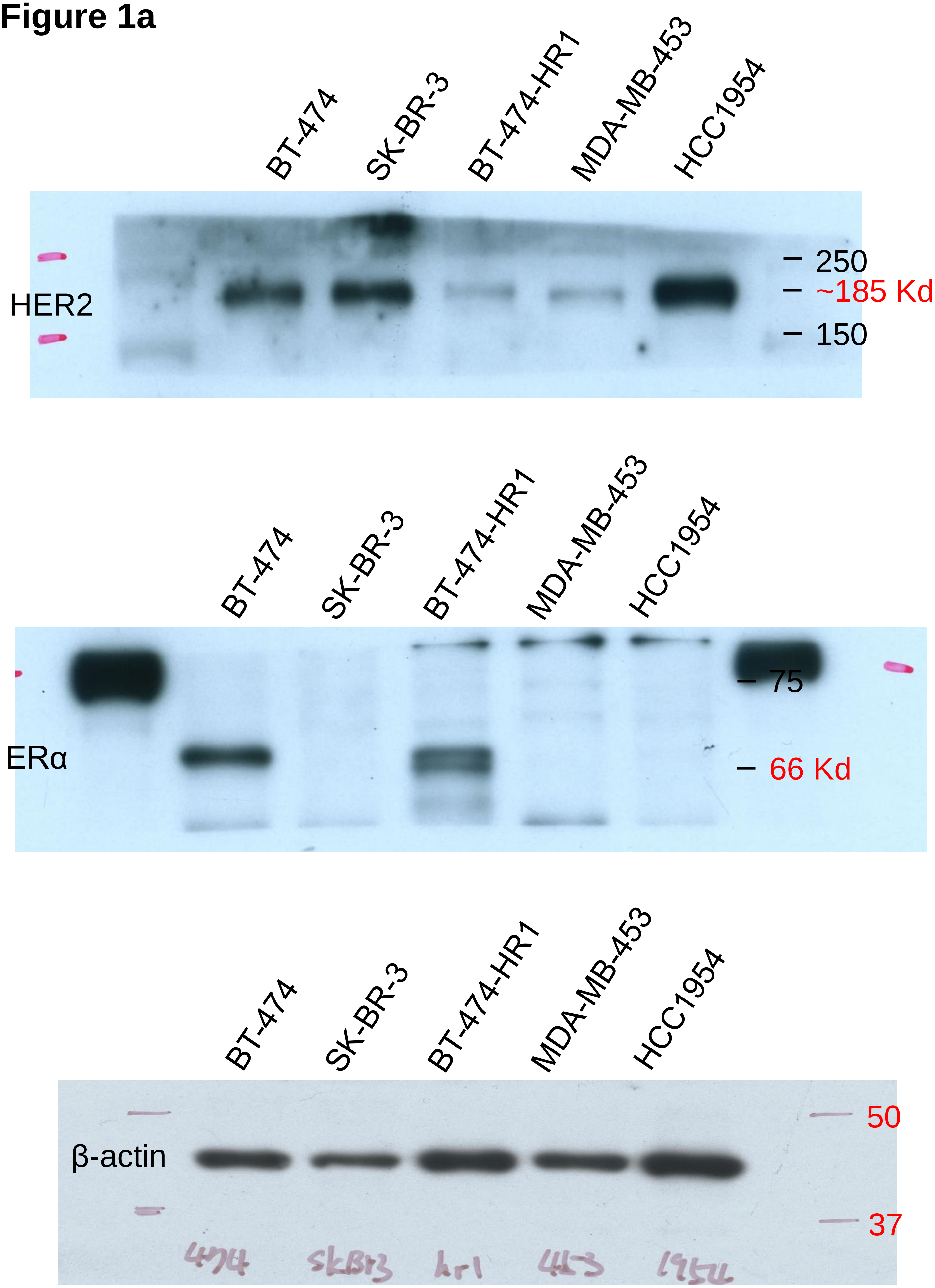
BT-474
SK-BR-3
BT-474-HR1
MDA-MB-453
HCC1954
HER2
250
~185 Kd
150
ERα
75
66 Kd
β-actin
50
37
474
SkBr3
hr1
453
1954

Figure 3a

BT-474-HR1

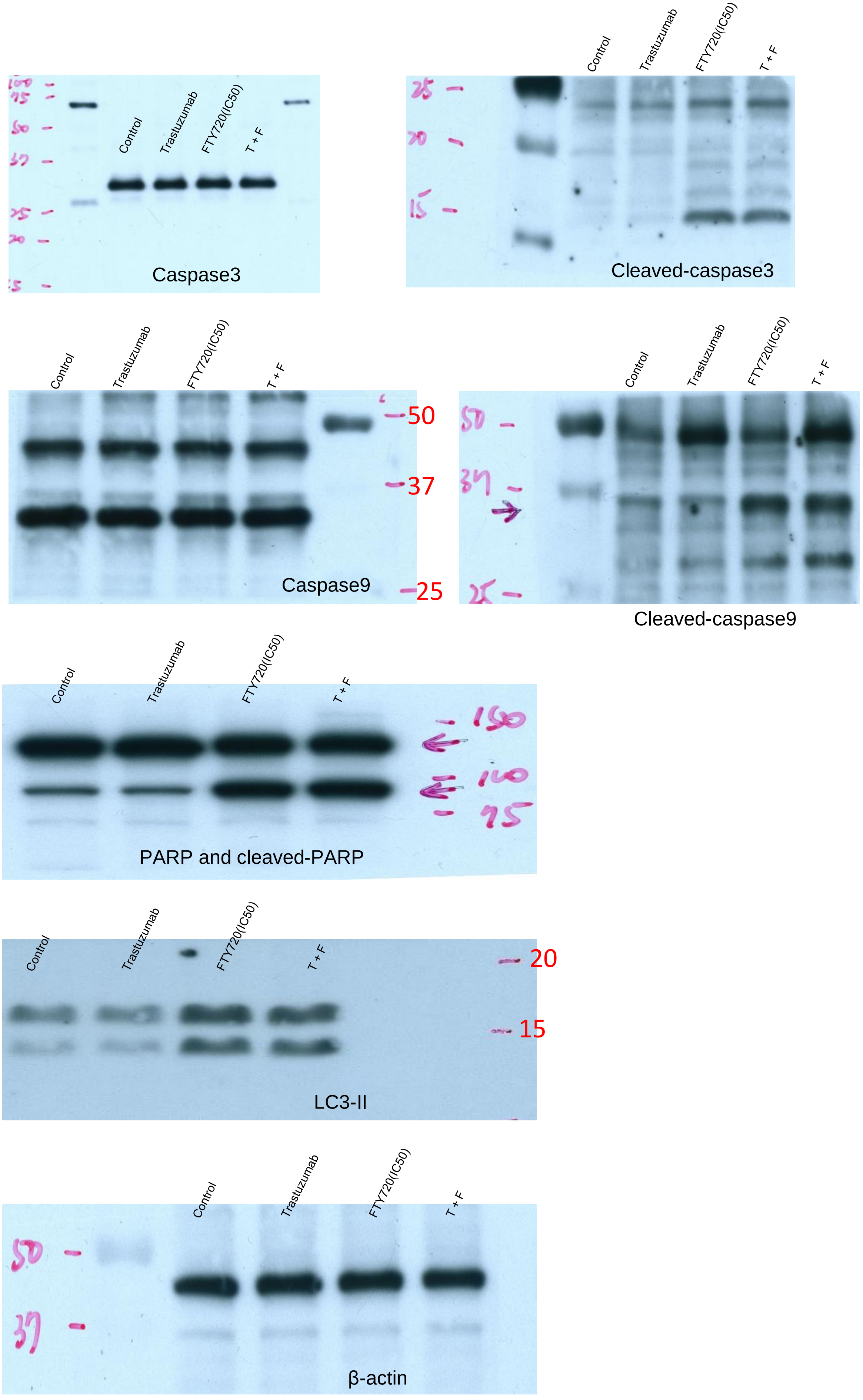

Figure 3a

MDA-MB-453

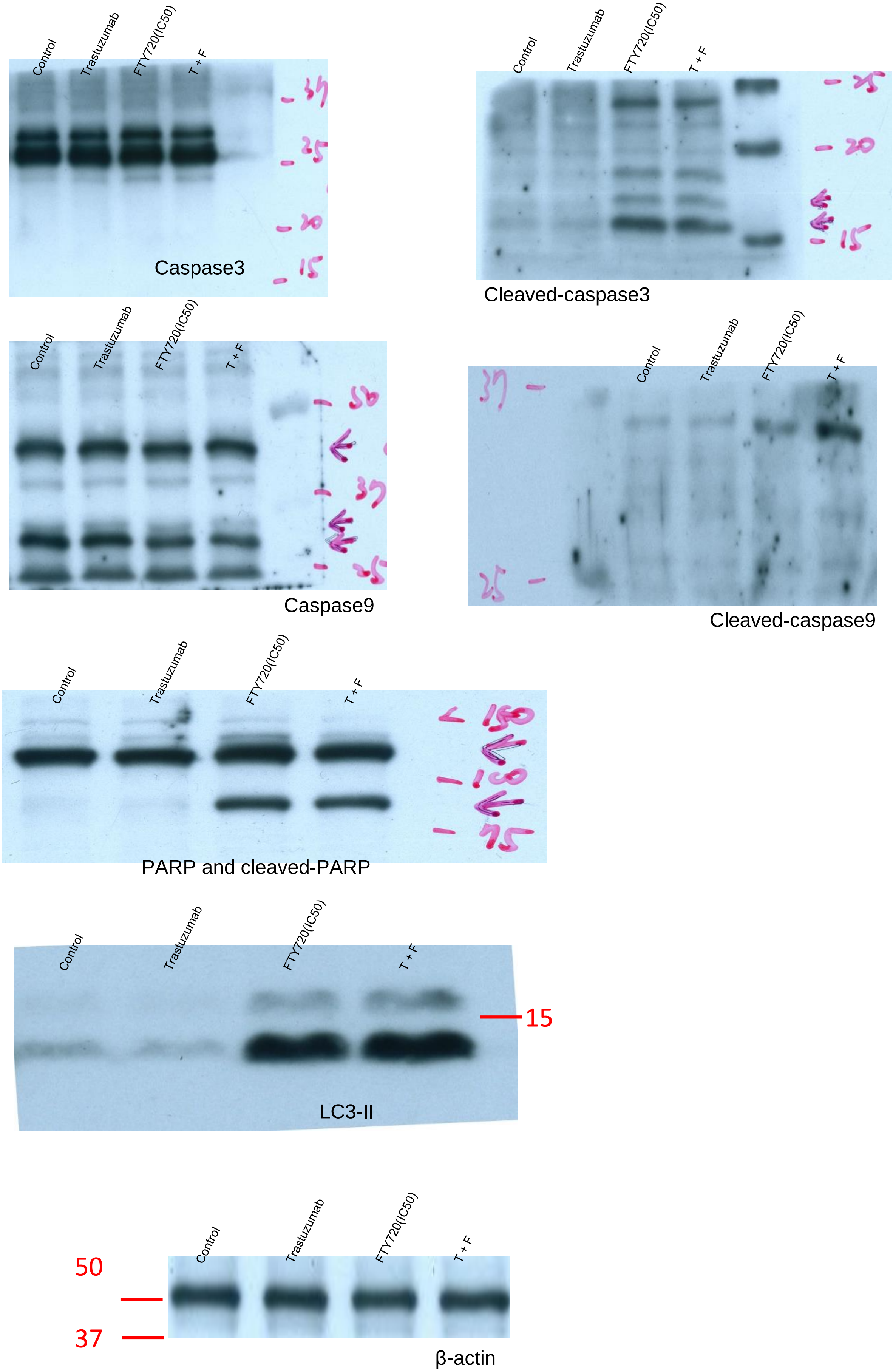

Figure 3a

HCC1954

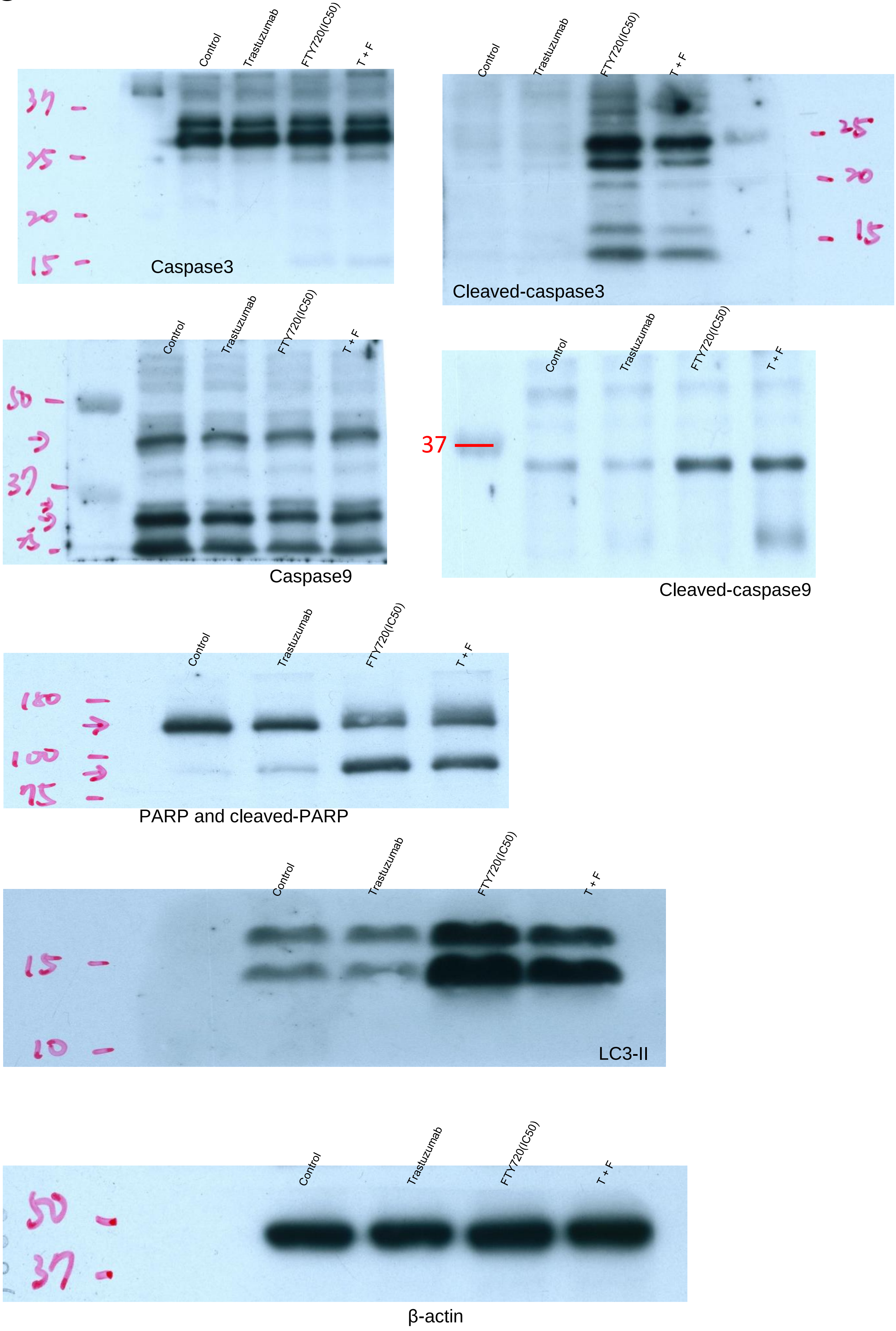

Figure 3b

BT-474-HR1

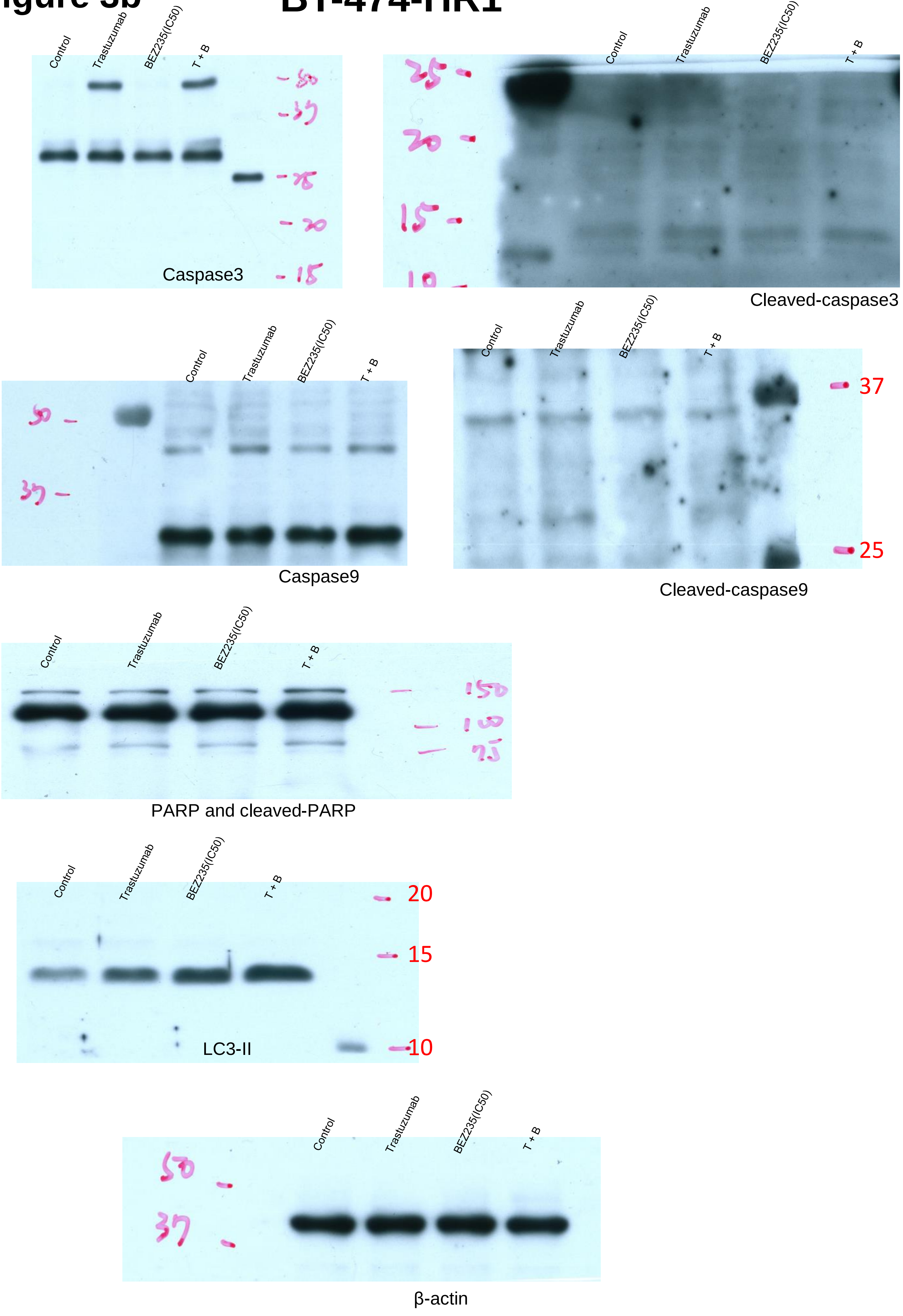

Figure 3b

MDA-MB-453

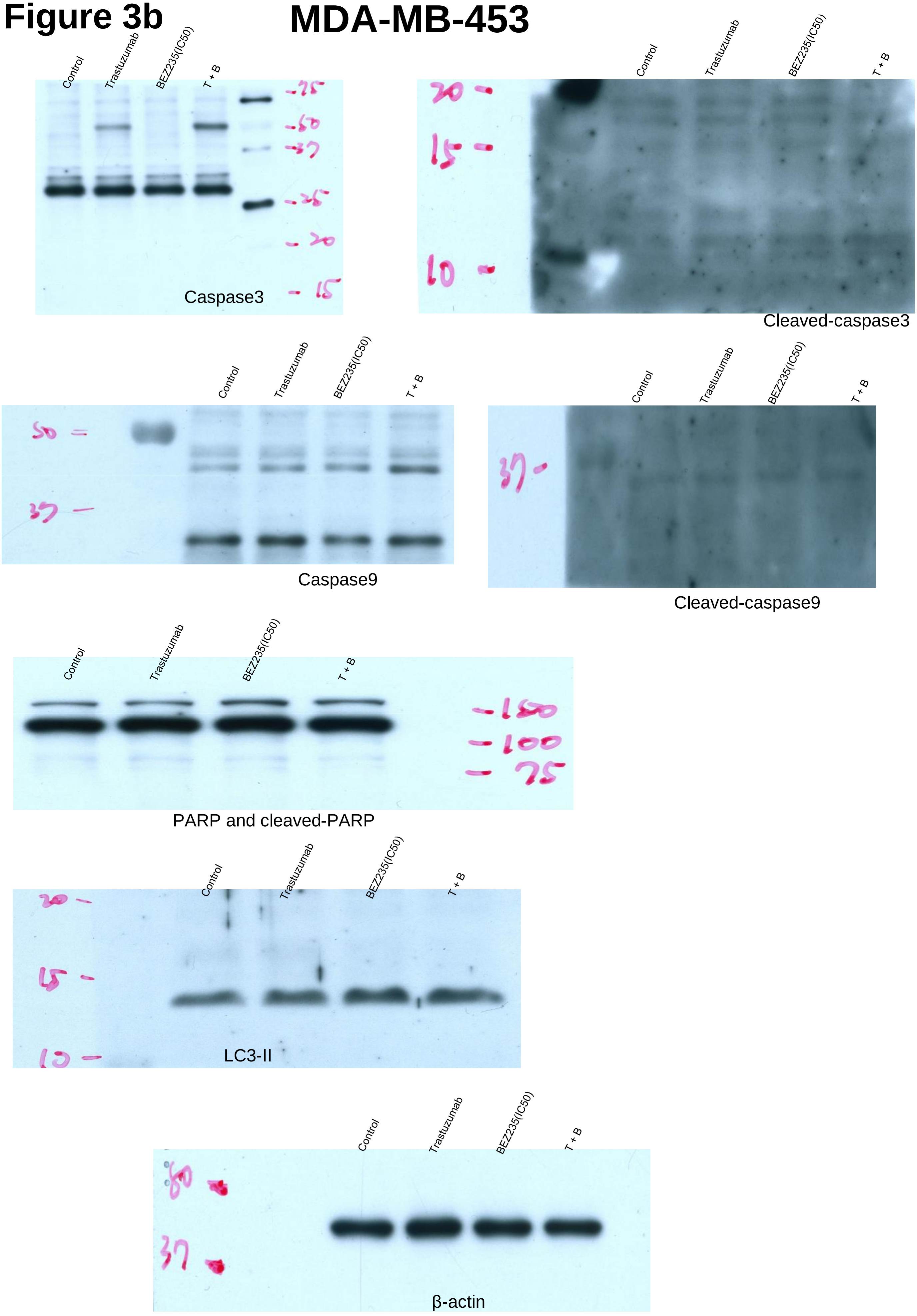

Figure 3b

HCC1954

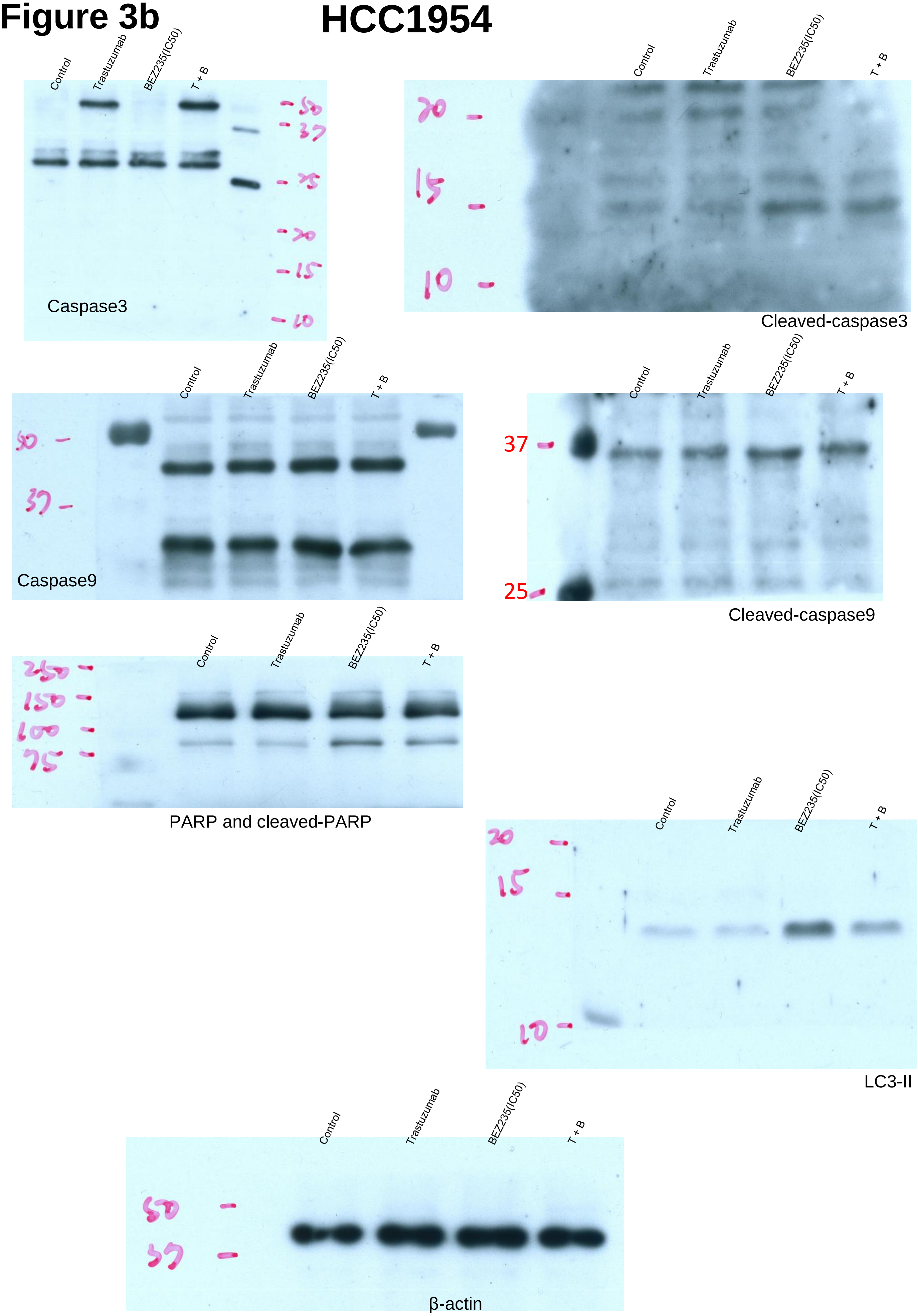

Figure 3c

HCC1954

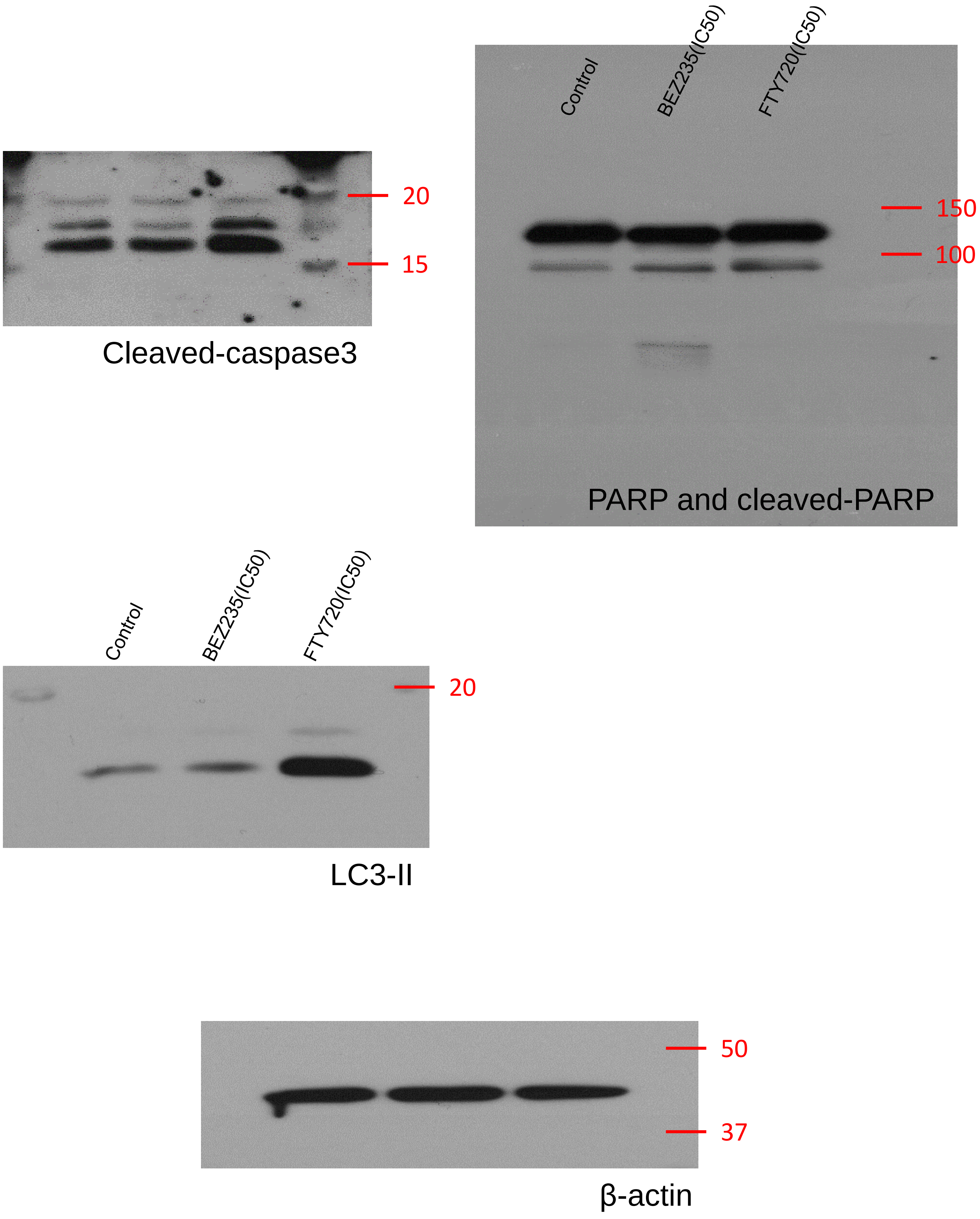

Figure 5b

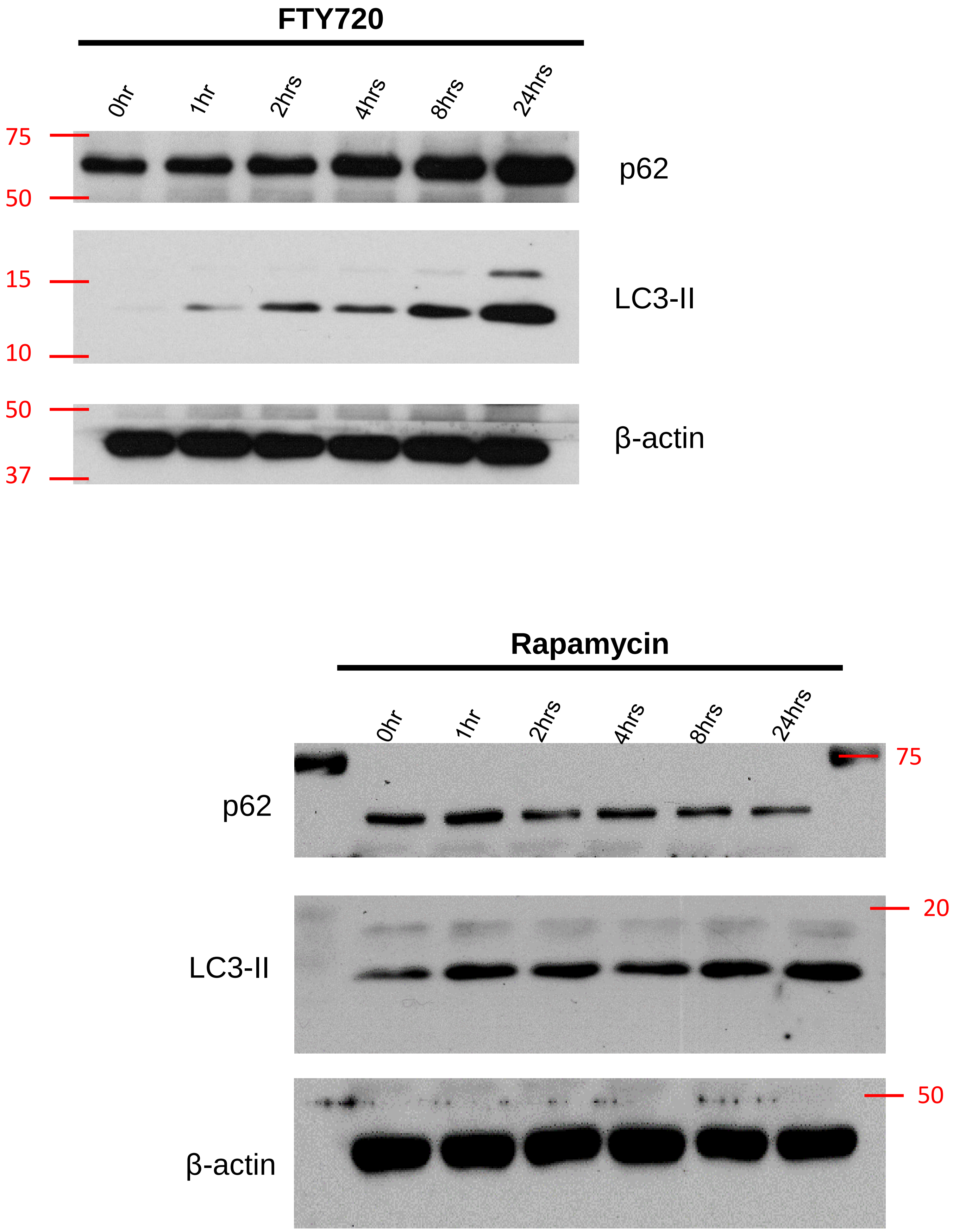

FTY720
0hr
1hr
2hrs
4hrs
8hrs
24hrs
75
50
p62
15
10
LC3-II
50
37
β-actin
Rapamycin
0hr
1hr
2hrs
4hrs
8hrs
24hrs
p62
75
LC3-II
20
β-actin
50

Figure 5b

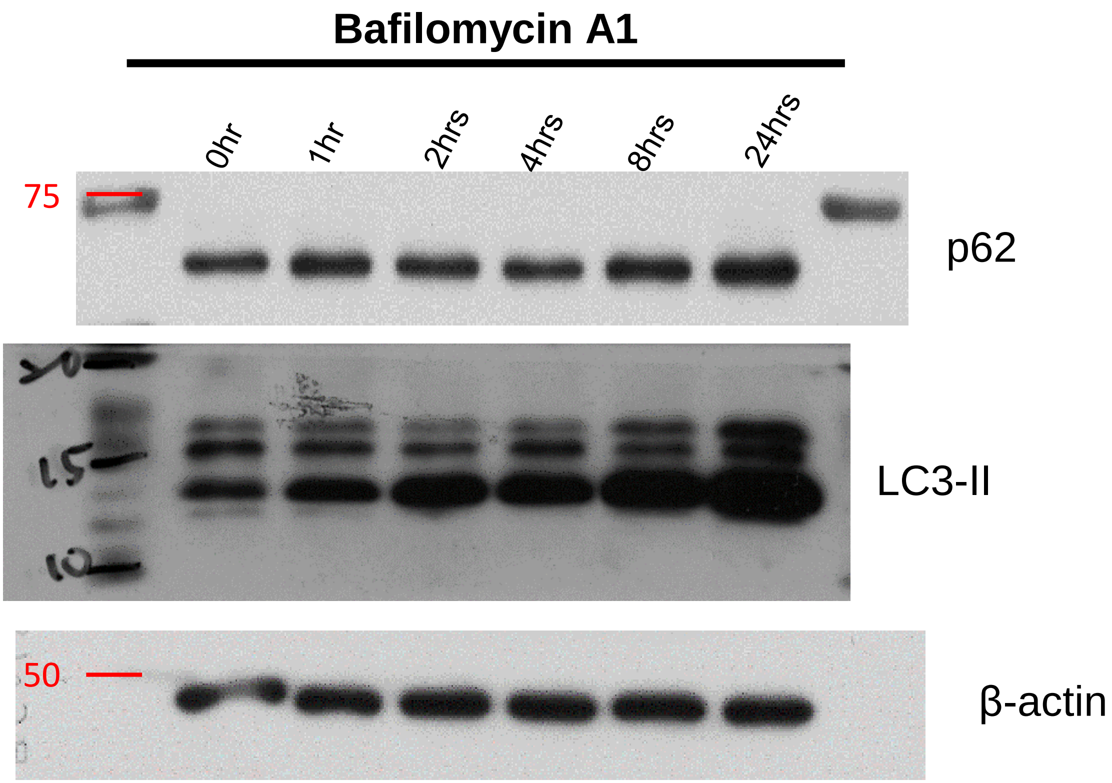
Bafilomycin A1
0hr
1hr
2hrs
4hrs
8hrs
24hrs
75
p62
15
10
LC3-II
50
β-actin

Figure 5c

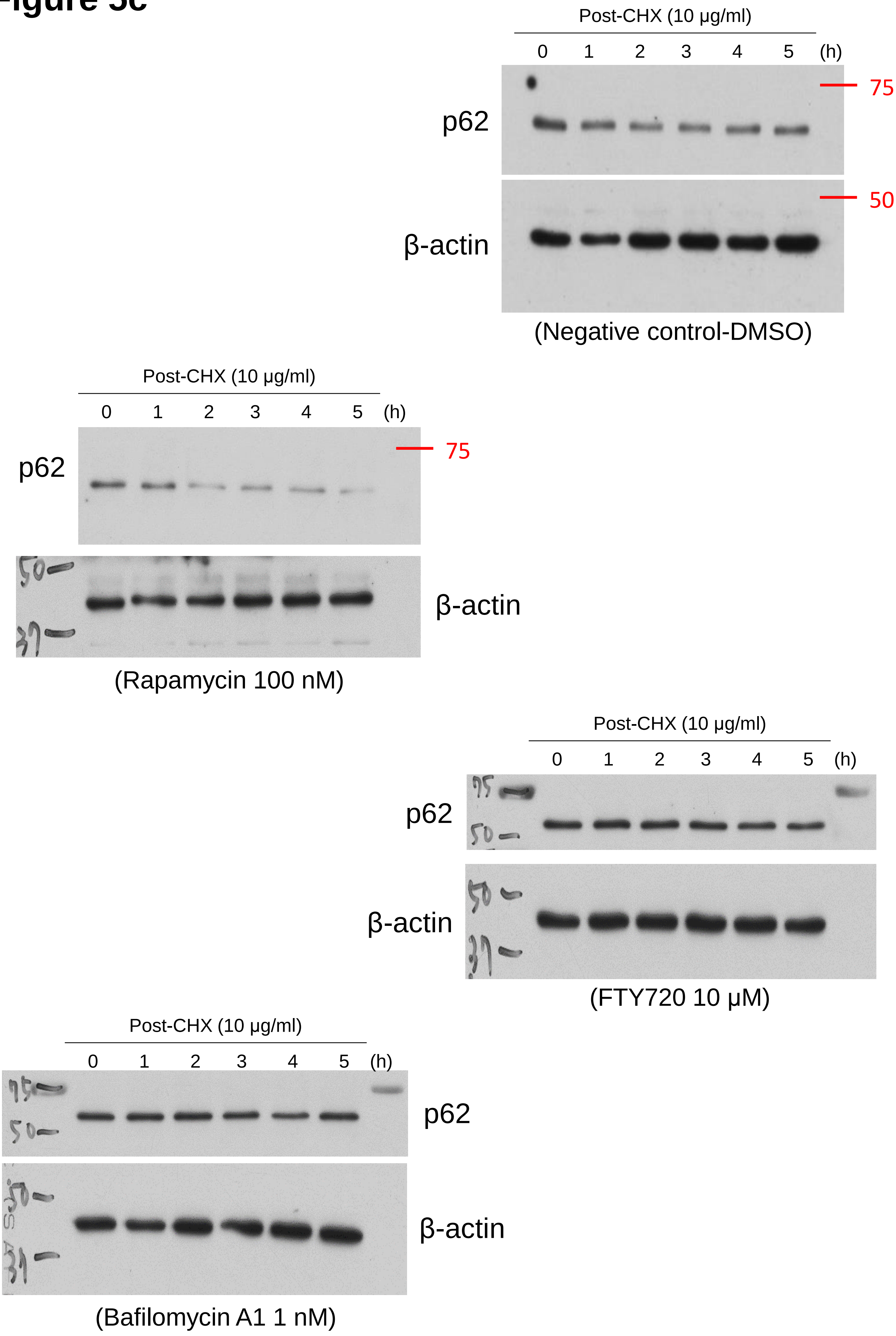
Post-CHX (10 µg/ml)
0 1 2 3 4 5 (h)
p62
75
β-actin
50
(Negative control-DMSO)
Post-CHX (10 µg/ml)
0 1 2 3 4 5 (h)
p62
75
β-actin
(Rapamycin 100 nM)
Post-CHX (10 µg/ml)
0 1 2 3 4 5 (h)
p62
β-actin
(FTY720 10 µM)
Post-CHX (10 µg/ml)
0 1 2 3 4 5 (h)
p62
β-actin
(Bafilomycin A1 1 nM)

Figure 5g

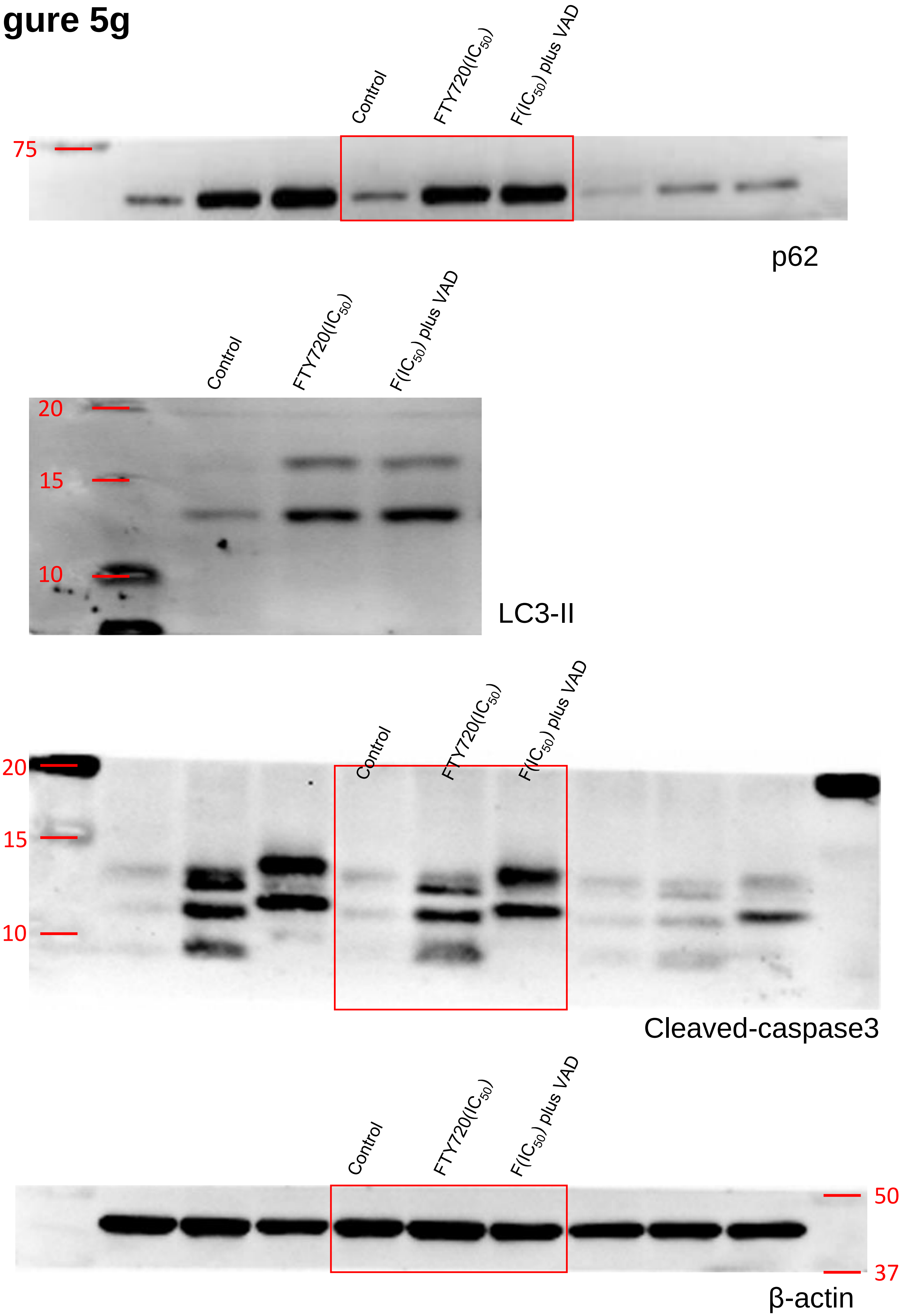
Control
FTY720(IC50)
F(IC50) plus VAD
75
p62
20
15
10
LC3-II
Cleaved-caspase3
50
37
β-actin

Figure 6c

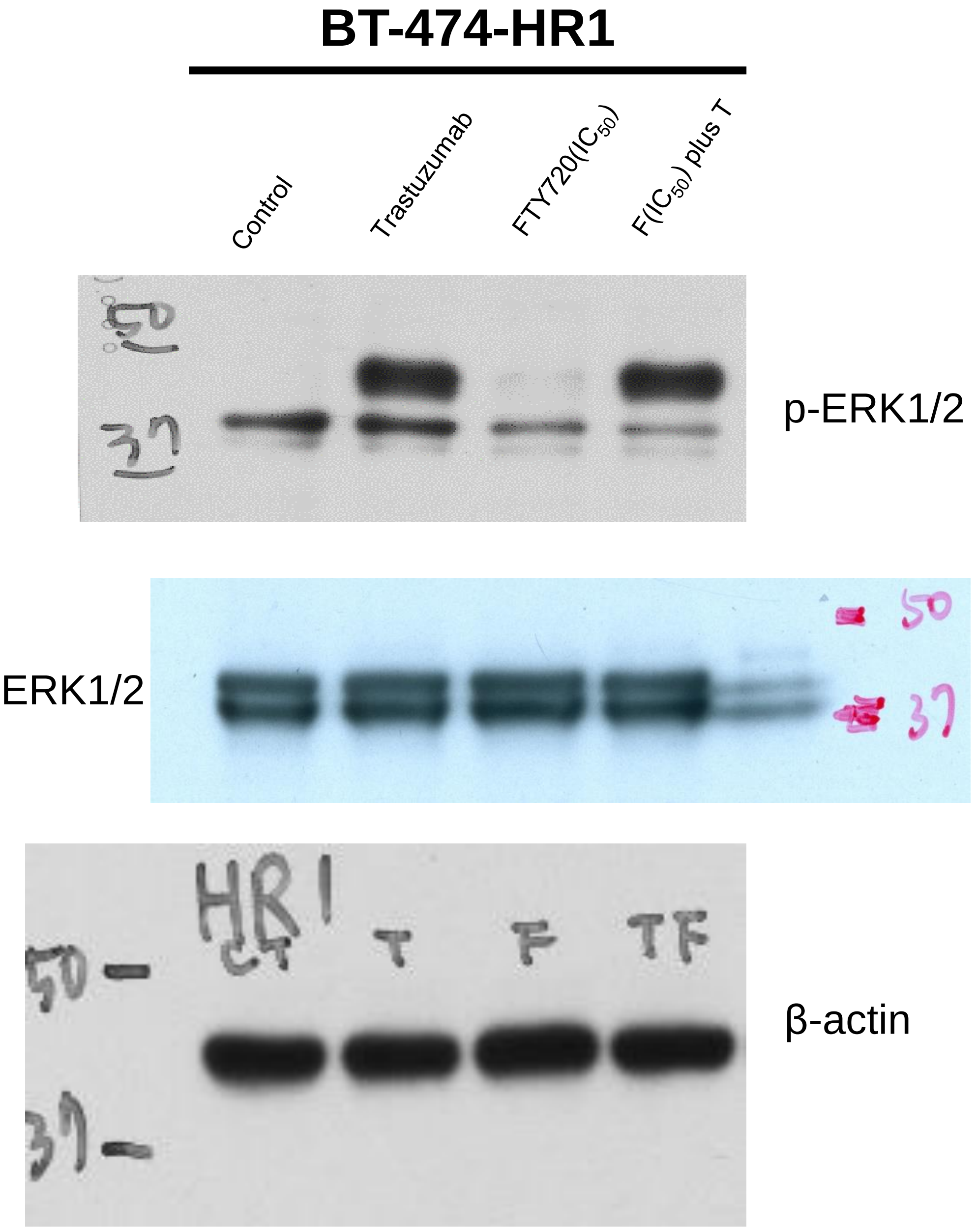

BT-474-HR1
Control
Trastuzumab
FTY720(IC50)
F(IC50) plus T
50
37
p-ERK1/2
ERK1/2
50
37
HR1
CT
T
F
TF
50
37
β-actin

Figure 6c

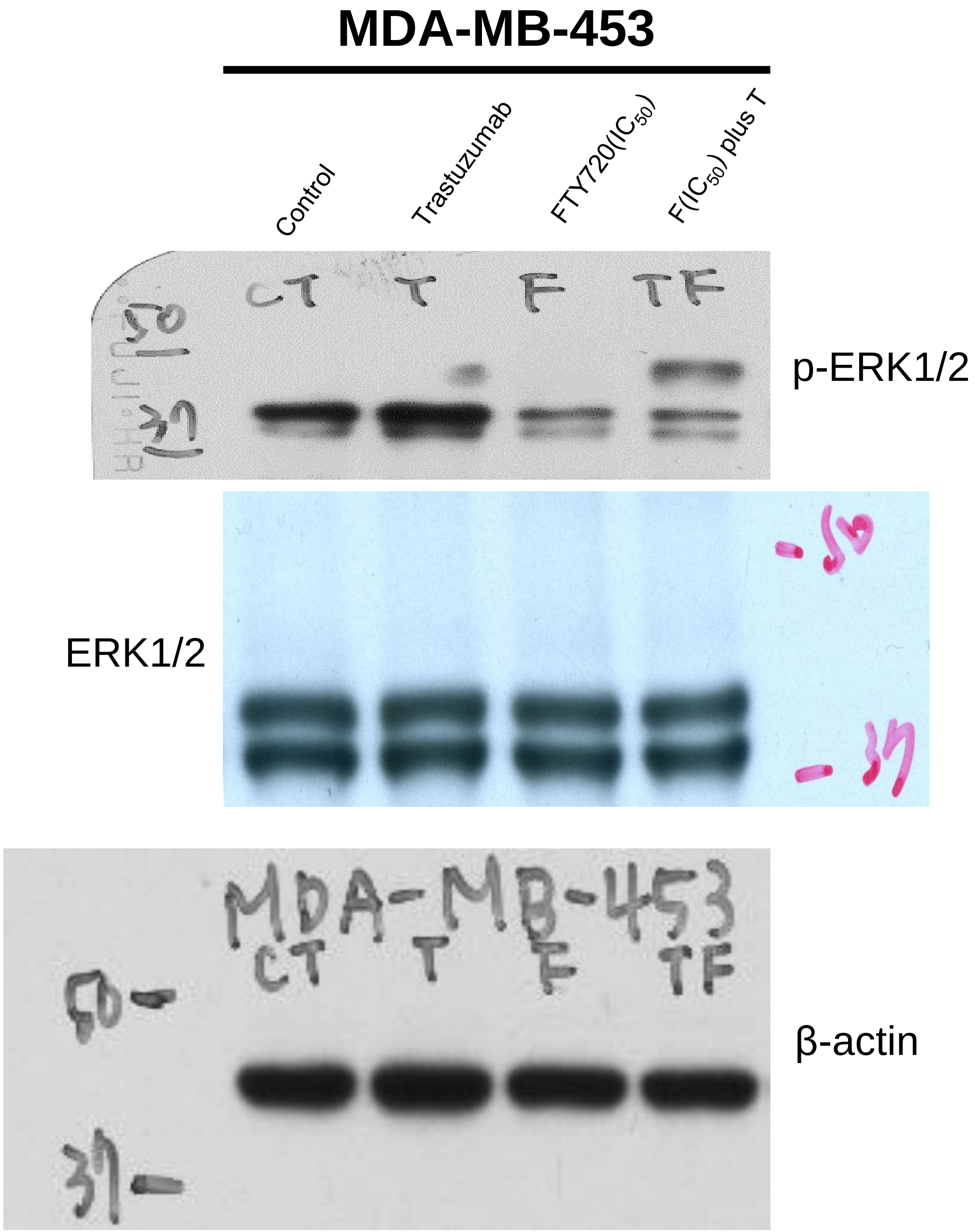
MDA-MB-453
Control
Trastuzumab
FTY720(IC50)
F(IC50) plus T
CT
T
F
TF
50
37
p-ERK1/2
ERK1/2
β-actin
MDA-MB-453

Figure 6c

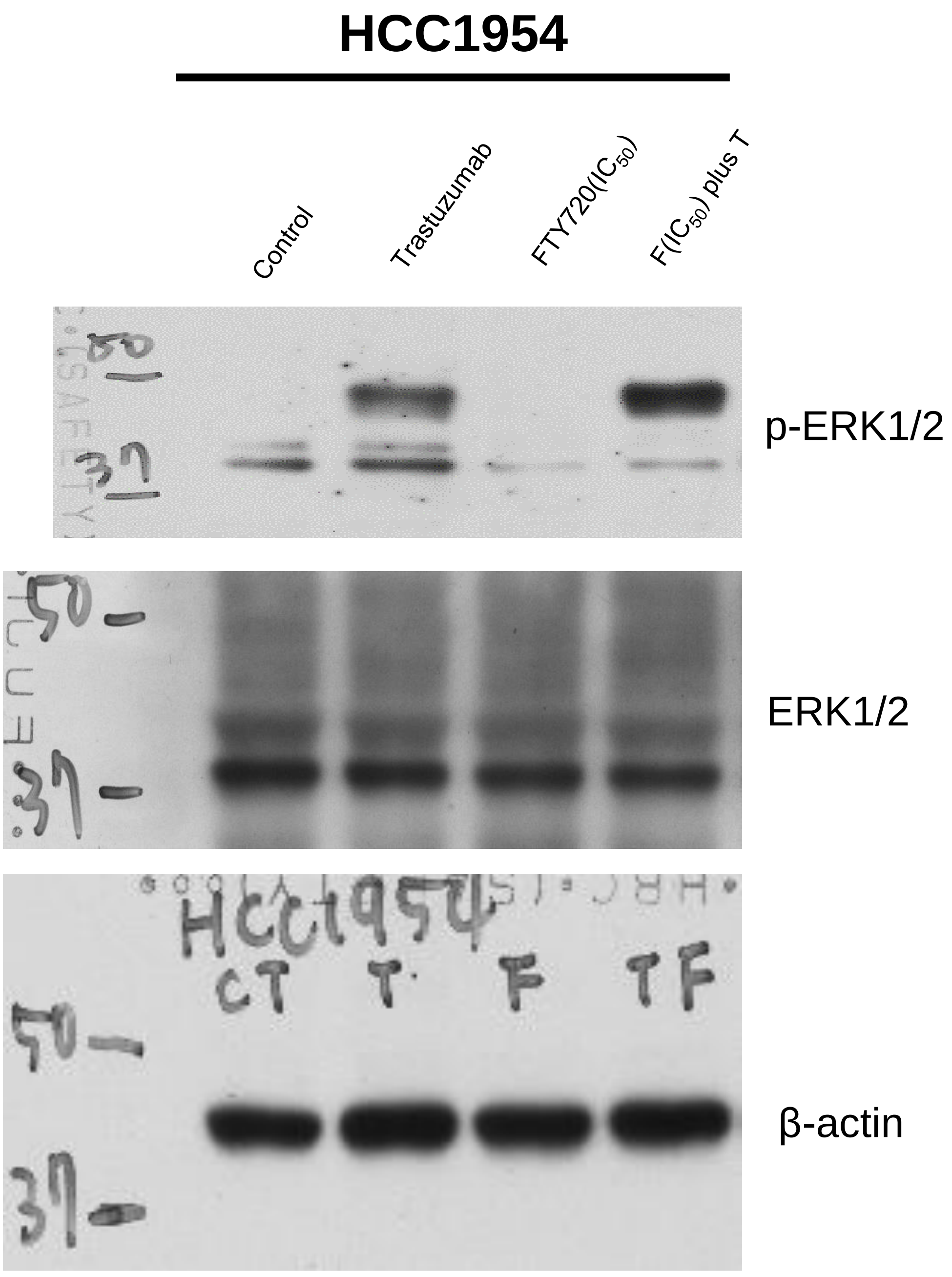
HCC1954
Control
Trastuzumab
FTY720(IC50)
F(IC50) plus T
p-ERK1/2
ERK1/2
β-actin
50
37
CT
T
F
TF
